# An improved crayfish optimization algorithm for solving engineering optimization problems

**DOI:** 10.1371/journal.pone.0340464

**Published:** 2026-02-26

**Authors:** Shuai Zhang, Shuai Zhang, Jinhuang You, Heming Jia, Chuanmin Wu, Chibiao Liu, Laith Abualigah

**Affiliations:** 1 School of Information Engineering, Fujian Key Lab of Agriculture IOT Application, Sanming University, Sanming, China; 2 Hourani Center for Applied Scientific Research, Al-Ahliyya Amman University, Amman, Jordan; SR University, INDIA

## Abstract

By emulating crayfish behaviors such as social foraging, rapid retreat from threats, and adaptive sensing, the Crayfish Optimization Algorithm (COA) achieves a dynamic balance between global search and local exploration, improving optimization efficiency. However, COA suffers from diversity degradation, insufficient exploration capability, low optimization finding accuracy, and easy fall to a local minimum. To solve these problems, an improved crayfish optimization algorithm (ICOA) is proposed. Firstly, the position of the population is achieved through the application of the Sobol sequence mapping in the initialization phrase, which enhances the diversity within the population. Secondly, a Lévy flight strategy is proposed in the foraging phase, which avoids algorithms fall into local optimization and enhances the individuals’ capacity for extensive exploration within the solution space. Subsequently, during the competition phase, using the Euclidean distance-fitness balanced competition strategy improves simultaneous development and exploration performance. To evaluate ICOA performance, the IEEE CEC2019 and CEC2020 benchmark functions and experiments were used in different dimensions for verification, followed by sensitivity analysis, quantitative analysis, and nonparametric statistical analysis. Furthermore, the effectiveness is validated in five engineering optimization problems, in which ICOA improved by 0.28%, 17.86%, 0.01%, 88.8% and 0.1%, respectively, compared to COA. ICOA exhibits enhanced optimization capabilities to tackle complex spatial and practical challenges. Incorporating multiple strategies markedly improve the efficacy of ICOA. This finding has significant implications in the field of engineering optimizations.

## 1 Introduction

With the increasing prominence of AI technology, many scholars show great interest in intelligent optimization [1,2], and the establishment of precise mathematical models to address complex optimization problems. Inspired by biological habits, physical phenomena, mathematical methods and other meta-inspired intelligent algorithms [3,4] are widely used in solving engineering optimization problems [3–5] such as hydrological Modeling and Prediction [6] and medical imaging area [7]. There are multiple design constraints in real-world applications, such as those being nonlinear, complex, and non-microscopic in nature, making them difficult to resolve. Facing these challenges, the prevailing solutions can be categorized into two types. The first category is deterministic algorithms. These algorithms leverage analytical characteristics and produce a deterministic sequence of points in globally optimal solution. These algorithms rely on precise mathematical formulations and follow a predictable trajectory toward the optimal solution. These deterministic methods include gradient descent, Newton’s method, and conjugate gradient, which are particularly effective for convex and differentiable functions. This type of algorithm is stable and predictable and often used in scientific computation, engineering design and other fields that require precise control and handling of deterministic problems. However, this type of algorithm can’t be applied to solve large-scale data and complex problems. The other category is metaheuristic algorithms [8–11], which are improvements on heuristics algorithms, combining the characteristics of local search algorithms and stochastic algorithms [12,13]. The standard meta-heuristic algorithms include Evolution-based, Physics-based, Human-based and Swarm-based algorithms. These algorithms are inspired by natural processes and are widely used for solving complex, non-linear and multi-modal optimization problems. An overview of various metaheuristic algorithms and their categories is presented in Table 1 and summarized as follows.

**Table 1:** The classification of meta-heuristics.

Evolution-based algorithm	Genetic Algorithm (GA) [14]
	Evolutionary Strategy (ES) [15]
	Evolutionary Programming (EP) [16]
	Genetic Programming (GP) [17]
Physics-based algorithm	Sine Cosine Algorithm (SCA) [18]
	Multi-Verse Optimization (MVO) [19]
	Simulated Annealing (SA) [20]
	Gravitational Search Algorithm (GSA) [21]
	Simulated Annealing (SA) [22]
	Snow Ablation Optimizer (SAO) [23]
	Social Network Search (SNS) [24]
Human-based algorithms	Running City Game Optimizer (RCGO) [25]
	Social Evolution And Learning Optimization Algorithm (SELOA) [26]
	Group Teaching Optimization Algorithm (GTOA) [27]
Swarm Intelligence-based algorithm	Wind Driven Algorithm (WDO) [28]
	Dwarf Mongoose Optimization (DBO) [29]
	Whale Optimization Algorithm (WOA) [30]
	Harris Hawks Optimization (HHO) [31]
	Particle Swarm Optimization (PSO) [32]
	Sand Cat Swarm Optimization (SCSO) [33]
	Crayfish optimization algorithm (COA) [34]
	Reptile Search Algorithm (RSA) [35]
	Horned Lizard Optimization Algorithm(HLOA) [36]
	Artificial Lemming Algorithm(ALA) [37]
Ancient-based algorithm	Giza Pyramids Construction(GPC) [38]
	Metaheuristic Search Optimization Algorithm(MSOA) [39]
Chemistry-based algorithm	Artificial chemical process(ACP) [40]
	Crystal Structure Algorithm (CryStAl) [41]
Plant-based algorithm	waterwheel plant technique(WWPA) [42]
	Willow Catkin Optimization(WCO) [43]
Music-based/Art-based algorithm	Harmony Search Algorithm(HSA) [44]
	Stochastic Paint Optimizer [45]
Sport-based algorithm	Running City game optimizer(RCGO) [46]
	Alpine Skiing Optimization [47]
Mathematical-based algorithm	Sine-Cosine Algorithm(SCA) [47]
	Stochastic Fractal Search(SFS) [48]
	Arithmetic Optimization Algorithm(AOA) [49]
	Tabu Search(TS) [50]
	Variable Neighbourhood Search(VNBS) [51]
Hybrid algorithm	Differential Evolution(DE) and Particle Swarm Optimizatiom(PSO) [52]
	Ant Colony Optimization(ACO) and Simulated Annealing(SA) [53]
	Cuckoo Search(CS) and Genetic Algorithm(GA) [54]

The crayfish optimization algorithm [34] simulates the behavior of the crayfish to forage, complete and avoid heat in the natural environment. It has a rapid convergence rate, which allows for a thorough search in the solution space and increases the possibility of finding the best global solution. However, it is more sensitive in parameter selection, and different problems may require different parameter settings, so its local search ability may be insufficient, which affects the search accuracy. In some complex optimization problems, the algorithm may experience premature convergence, resulting in the inability to find all optimal solutions. When simulating behaviors and position updates, it requires larger individuals and may require more computational resources to address large-scale issues.

The optimization research on crayfish at home and abroad focuses mainly on three aspects. The first is the improvement algorithm. Researchers are committed to improving the performance of crayfish optimization algorithms, including increasing convergence speed, improving overall search ability, avoiding premature convergence, and so on. Chao [55] proposed an advanced artificial intelligence-based method that uses a parallel Crayfish Optimization and Arithmetic Optimization Algorithm (PSCOAAOA) to optimize the parameters of a Support Vector Machine (SVM), allowing the prediction of the friction force for the Ti-6Al-4V alloy under different lubrication conditions. Wang [56] introduces strategies such as cave candidacy, food covariance learning, and non-monopoly perturbation to address the shortcomings of the original algorithm. Secondly, in theoretical research, some scholars focus on the theoretical foundations of the crayfish algorithm, including convergence analysis, complexity analysis .etc. Through theoretical research, a further understanding of the characteristics and adaptation range of the algorithm. Shikoun [57] enhances the original COA with refracted opposition-based learning and a crisscross strategy to improve search ability and convergence accuracy. Zhang [58] proposed an enhanced crayfish optimization algorithm to improve the exploration capability and avoid being caught in local optima. Third, COA can be applied to industrial production scheduling, image processing, path planning, medical, and other practical problems. Chaib [59] uses fractional-order chaos maps in COA, then adaptively adjusts COA settings based on energy requirements. Jia [60] combines a learning strategy with COA strengthening the algorithm’s ability to explore the solution space and escape local minima. Patel [61] proposes an intelligent kidney tumor segmentation and classification model for the early identification of benign and malignant tumors based on a deep learning model and MCOA. This proposed method significantly improves the accuracy and efficiency of tumor detection. Xiao [62] combines specular reflection learning, the expanded exploration strategy from the Aquila optimizer(AO), the local exploitation characteristics of Lévy flight, and a vertical crossover operator to enhance its optimization capability.

COA suffers from diversity degradation, insufficient exploration capability, low optimization finding accuracy, and easily falling into a local minimum. To optimize these limitations, this paper proposes an ICOA. First, use the Sobol sequence to intialize the population, which can effectively make its distribution average. Second, to balance global and local exploration in the competition phase, a Euclidean distance-fitness balanced competition strategy is proposed. Finally, the position of the crayfish is randomly updated using the Lévy flight strategy, with additional update conditions incorporated to accelerate the convergence rate. The optimization performance of ICOA is evaluated in CEC2019 and CEC2020. The main contributions of this study are summarized as follows.

- Use the Sobol sequence to initialize the population, which can effectively make its distribution average in the initial stage.
- To balance exploration and exploitation during the competition phase, a Euclidean distance-fitness balanced competition strategy is proposed in the competition phase.
- To enhance global optimal search ability, the position of the crayfish was randomly updated using the long-short jump feature of theLévy’s flight.
- ICOA is evaluated on 10 functions in CEC2019 and CEC2020, which utilize various dimensions.
- ICOA is evaluated in five engineering applications.

The overall research framework of this paper is organized as follows:

The Sect 2 reviews the classical COA and discusses the motivations for improving its search mechanisms. The Sect 3 presents improvements from three perspectives: initialization improvements, exploration strategies, and hybrid COA variants. The Sect 4 conducts a performance analysis on the CEC2019 and CEC2020 benchmark functions in different dimensions, and evaluation metrics. Reports and analyzes the comparative results, followed by Sensitivity analysis, statistical tests and ablation studies. The Sect 5 validates the effectiveness of the proposed method through five engineering optimization problems. Finally, the Sect 6 concludes the paper by discussing the advantages, limitations, and future research directions of the proposed algorithm.

## 2 Crayfish optimization algorithm

COA simulates key behavior stages of crayfish, including escaping summer heat, engaging in competition, and searching for food.. The summer heat avoidance stage is exploration stage, and competition phase and foraging behaviour stage is its development stage. The whole algorithm has a more organic optimization effect due to the temperature balancing algorithm exploration and development capability, which can find the best fitness value more quickly.

### 2.1 Initialization stage

In COA, every individual crayfish is set as a matrix structure, in a set of variables $\left(X_{1,1},X_{1,2},\ldots ,X_{1,dim}\right)$, a set of potential solutions *X* will be created randomly. Additionally, each variable should remain within its respective boundary limits.

$$X=\left[X_{1},X_{2},\ldots ,X_{N}\right]=\left[\begin{array}{c} \begin{array}{c} X_{1,1}\;\;\cdots \;\;X_{1,j}\;\;\cdots \;\;X_{1,\text{dim}}\\ \vdots \;\;\cdots \;\;\vdots \;\;\cdots \;\;\vdots \\ X_{i,1}\;\;\cdots \;\;X_{i,j}\;\;\cdots \;\;X_{i,\text{dim}}\\ \vdots \;\;\cdots \;\;\vdots \;\;\cdots \;\;\vdots \end{array}\\ X_{N,1}\;\;\cdots \;\;X_{N,j}\;\;\cdots \;\;X_{N,\text{dim}} \end{array}\right]$$ (1)

$$X_{i,j}=Lb_{j}+(Ub_{j}-Lb_{j})\times \text{rand}$$ (2)

where, *N* represents the total number of crayfish in the population, *dim* represents its dimension, *X* is the initial population, *X*_*i*,*j*_ represents the coordinate of the *i*-th crayfish along the *j*-th axis, *Ub*_*j*_ means the upper boundary in the *j*-th dimensional, *Lb*_*j*_ is lower boundary in the *j*-th dimension, rand means a random value in the range [0,1].

### 2.2 Summer heat avoidance stage (exploratory phase))

Temperature Changes will affect the behavior of the crayfish. When the temperature exceeds a certain value, the crayfish will seek cooler areas to escape the heat. The optimal feeding temperature of crayfish is in the range[20,30]. The temperature of crayfish is given by Eq 3:

tmp=rand×15+20 (3)

In the summer heat avoidance stage (exploratory phase), if it exceeds a certain value, the crayfish will move into a cave to escape the heat. Subsequently, they will select a relatively cooler location to avoid exposure to high temperatures. The mathematical model for this heat avoidance behavior in crayfish is presented in Eq 4:

$$\left[X_{shade}=\frac{X_{G}+X_{L}}{2}\right]$$ (4)

Where *X*_*G*_ refers to the best position achieved during multiple iterations. and *X*_*L*_ refers to the optimal position obtained after the update of the current iteration. The competition among crayfish for the cave occurs randomly. In the summer heat avoidance stage (exploratory phase), when *rand* < 0.5, if there are no competing crayfish, it enters the cave directly to escape the heat. The updated position is set in the following Eq 5:

$$X_{new}=X_{i,j}+C_{2}\times rand\times \left(X_{shade}-X_{i,j}^{t}\right)$$ (5)

Here, *X*_*new*_ is the post-update position used for the subsequent generation, and *C*_2_ follows a decreasing trend from 2 to 0. *C*_2_ is calculated in the following Eq 6:

$$C_{2}=2-\left[\frac{FEs}{MaxFes}\right]$$ (6)

### 2.3 Competition stage (exploitation stage)

The crayfish begin to compete to the cave once the temperature >30^°^C and rand⩾0.5. In this situation, the crayfish *X*_*i*_ updates the position based on another randomly selected crayfish *X*_*z*_. The updated position of the crayfish is calculated using Eq 7:

$$X_{new}=X_{i,j}-X_{z,j}+X_{shade}$$ (7)

Here, *X*_*z*,*j*_ is the position of a random crayfish, and the formula for calculating the random crayfish is presented in Eq 8.

z=round(rand×(N−1))+1 (8)

### 2.4 Foraging stage (exploitation stage)

The crayfish will come out of their cave to search for food once the temperature$⩽30^{\circ }C$. In Addition, temperature plays a role in determining how much food the crayfish will eat. The crayfish have a strong foraging behavior when the temperature is between $25^{\circ }C$ and $30^{\circ }C$. And when the temperature is equal to $25^{\circ }C$, crayfish will find the most food. The food intake of crayfish depends on the temperature and is normally distributed. Food intake is calculated in the following Eq 9:

$$p=C_{1}\times \frac{1}{\sqrt{2\times \pi \times \sigma }}\times \exp\left(-\frac{(temp-\mu {)}^{2}}{2\sigma^{2}}\right)$$ (9)

The position of the food is calculated according to the Eq 10:

$$X_{food}=X_{G}$$ (10)

*temp* is the environmental temperature, *μ* is the best temperature for crayfish to eat, *σ* and *C*_1_ are factors influencing food intake adjustments based on temperature variations.

When eating, crayfish decide whether to split the food apart depending on their size. When the food is the right size, they will eat it immediately. Nevertheless, if the food is too large, they will split it into smaller pieces before eating it. The size of the food is evaluated using the following Eq 11.

$$Q=C_{3}\times rand\times \left(\frac{{\text{fitness}}_{i}}{{\text{fitness}}_{food}}\right)$$ (11)

Here, *C*_3_ is a food-related parameter associated with the maximum food size. ${\text{fitness}}_{food}$ represents the fitness of the location of the food, while ${\text{fitness}}_{i}$ is the fitness value of the *i*-th crayfish.

If *Q*>(*C*_3_  +  1)/2, the crayfish cannot directly eat the food due to its size. When the food is too large, the crayfish first breaks it into pieces with its claws, then uses its second and third pairs of legs in turn to feed. The formula for slicing the food is presented in Eq 12.

$$X_{food}=\exp\left(-\frac{1}{Q}\right)\times X_{food}$$ (12)

The new feeding position is computed using the formula shown in Eq 13:

$$X_{new}=X_{i,j}+X_{food}\times p\times (\cos(2\times \pi \times rand)-\sin(2\times \pi \times rand))$$ (13)

If *Q*<(*C*_3_ + 1)/2, since the food is of a suitable size, the crayfish immediately goes to it and begins eating without the need for prior processing. The new feeding position is given in Eq 14:

$$X_{new}=(X_{i,j}-X_{food})\times p+p\times \text{rand}\times X_{i,j}$$ (14)

### 2.5 Pseudo-code of the COA

**Fig 1:**
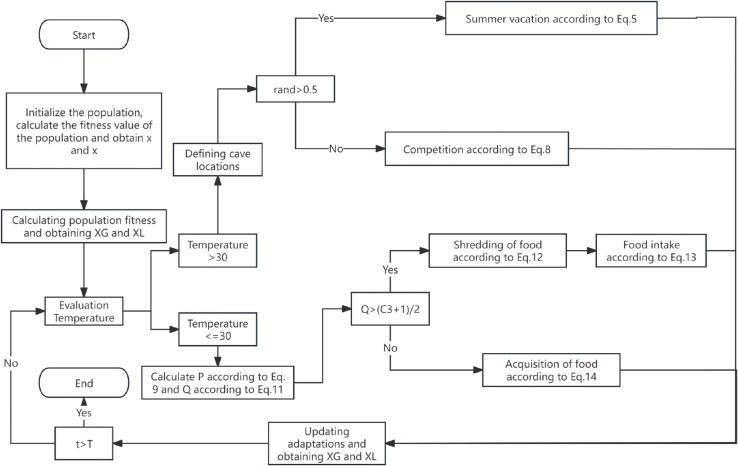
The flow diagram of COA.

The COA pseudo-code is detailed in Algorithm 1 and Fig 1.

**Algorithm 1 Pseudo-code of the COA.**

  Initialization: Population *Np*, Iteration *T*, Dimension *Dim*; Initialize the population, evaluate the fitness to identify *X*_*Gbest*_, *X*_*Lbest*_.

  **while** *t*<*T* **do**

   Calculate the temperature using Eq 3

   **if** temperature > 30 **then**

    Compute *X*_*shade*_ via Eq 4

    **if** rand < 0.5 **then**

     Adjust the location by using Eq 5

    **else**

     Adjust the location by using Eq 7

    **end if**

   **else**

    Evaluate the relationship between food intake proportion and food size using Eqs 9 and 11

    **if** Q > (C3 + 1)/2 **then**

     Adjust the location by using Eq 13

    **else**

     Adjust the location by using Eq 14

    **end if**

   **end if**

   **for** 1:N **do**

    Adjust fitness values, *X*_*Gbest*_, *X*_*Lbest*_

   **end for**

   *t* = *t* + 1

  **end while**

## 3 Improved crayfish optimization algorithm

### 3.1 Initialization improvements

Crayfish are highly territorial. In the natural environment, crayfish are evenly distributed at the bottom of the pool and cannot tolerate the presence of crayfish of the same sex within their burrows. This territoriality is manifested not only in the fact that they cannot tolerate the same kind in their territories. In addition, the size and location of the territories will be adjusted with changes in time and the ecological environment. In meta-heuristic optimization, how initial solutions are positioned within the solution space has a significant impact on both search accuracy and convergence speed. Uniformly initializing the population improves the algorithm’s search efficiency. COA employs a random generation method for the initialization of solutions, which leads to low traversability, slow convergence, and uneven population distribution. This paper employs the Sobol sequence for population initialization, and uniform points are used to fill the multidimensional hypercube as much as possible, which results in higher computational efficiency and broader coverage of sampling points.

**Fig 2:**
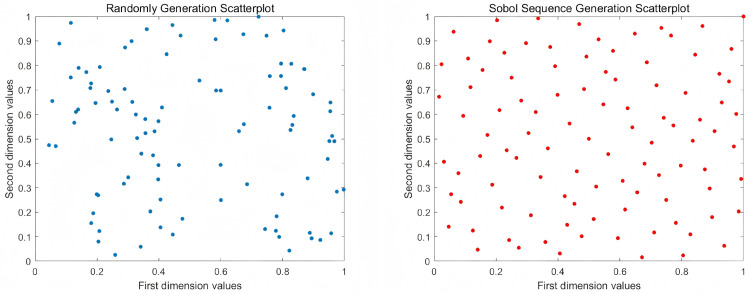
Randomly generated and Sobol sequence generation scatterplot comparison graph.

The sobol sequence is an efficient random-number generation method with low variance and produces random search directions. In each dimension of the population, there exists a Radical Inversion with a base number of 2, and each dimension of the Radical Inversion possesses its distinct matrix, thereby generating non-repetitive and uniform points. Fig 2 shows randomly generation and Sobol sequence generation scatterplot comparison graph, the random number distribution of a population of 100 sizes in the interval of [0,1], which is compared with the random number distribution in the 2D space. Compared to other methods, the Sobol sequence produces more uniform populations, and the coverage of the solution space is more complete.

### 3.2 Exploration strategies

In the foraging phase, when $Q\leq (C_{3}+1)/2$, the crayfish experience slow convergence during direct food acquisition. COA may prematurely converge when search agents cluster too quickly, which motivates the incorporation of Lévy flights to allow long-distance jumps and escape from local optima. A modified conditional update optimization algorithm is introduced that incorporates the Lévy flight strategy. This algorithm utilizes the characteristics of the heavy-tailed distribution of Lévy flight to randomly adjust the position of the crayfish. Significantly enhances global search capabilities by combining frequent local steps with occasional long-distance jumps, allowing the algorithm to escape local optima and accelerate convergence. The expression of Lévy step size is defined in the following Eq 15:

$$s=\frac{u}{|v{|}^{\frac{1}{\beta }}}$$ (15)

Where:

- $u\sim N(0,\sigma_{u}^{2})$: a standard normal distribution with zero mean and variance $\sigma_{u}^{2}$.
- $v\sim N(0,\sigma_{v}^{2})$: a standard normal distribution with zero mean and variance $\sigma_{v}^{2}$.

The variance $\sigma_{u}$, $\sigma_{v}$ is calculated using Eq 16:

$$\{\sigma_{u}={\left[\frac{\Gamma (1+\beta )\sin(\frac{\pi \beta }{2})}{\Gamma (\frac{1+\beta }{2})\beta 2^{\frac{\beta -1}{2}}}\right]}^{\frac{1}{\beta }}\;\sigma_{v}=1$$ (16)

In Eq 16, Γ presents the standard gamma function, *β* represents the value range [0,2]. Here, the value is taken as 0.8.

When $Q\leq (C_{3}$  +  1)/2, crayfish immediately after eating food in the position update formula (Eq 14), thus allowing the algorithm to be more efficient in escaping from local optima. The new crayfish updating formula is presented in the following Eq 17:

$$X_{new}=(X_{i,j}-X_{food})\times p+p\times X_{i,j}\times \delta ⊗s$$ (17)

where, *δ* is the step scaling factor, set to 1. ⊗ denotes the operation of tensor product.

### 3.3 Hybrid COA variants

During the competition stage, the update strategy is designed primarily to promote a deeper exploration of the solution space. However, the initial formulation favors random exploitation and reduces the effectiveness of exploitation. And the original distance-update mechanism lacks adaptive sensitivity to the spatial relationships among individuals, motivating the use of an Euclidean-distance-based strategy to refine position update dynamics. This research presents a balanced competition strategy that incorporates both Euclidean distance and fitness metrics for agent selection. To ensure adequate exploitation performance throughout the competition phase while allowing agents to be ranked according to their fitness levels, we introduce a beta parameter that balances Euclidean distance with fitness. Subsequently, any agent from the top 50% is selected based on this balance. This approach effectively synchronizes exploration and exploitation performance more optimally. The following Eqs 18 to 24 show the modified competition phase formula:

$$\beta =1-e^{\frac{FEs}{MaxFEs}}$$ (18)

$$distance(i,j)=\sum_{k=1}^{dim}{\left(X_{i,k}-X_{j,k}\right)}^{2}$$ (19)

$$finess\_score=\frac{fitness}{fitness_{max}}$$ (20)

$$X_{score(i)}=\beta \times fitness\_score+distance(i)$$ (21)

$$X_{Edf}=Select\_Random{\left[X_{score}\right]}_{top50\%}$$ (22)

$$X_{new}=X_{i,j}-X_{Edf,j}+X_{shade}$$ (23)

$$\{X_{new}=X_{i,j}-X_{Edf,j}+X_{shade}\text{if rand}<0.3\;X_{new}=X_{i,j}-X_{z,j}+X_{shade}\text{else}$$ (24)

Where distance measures how far the current individual is from the other individuals in Euclidean terms. *fitness_score* is a 1×N matrix, *X*_*Edf*_ is the index of the selected individual.

### 3.4 Pseudo-code of the ICOA

**Fig 3:**
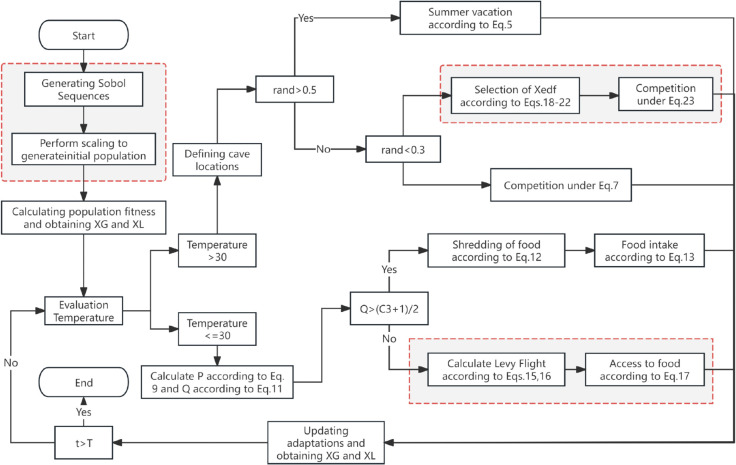
The flow diagram of ICOA.

The ICOA pseudo-code is detailed in Algorithm 2 and Fig 3:

**Algorithm 2 Pseudo-code of ICOA.**

  Initialization: Population *Np*, Iteration *T*, Dimension *Dim*; Initialize the population, evaluate the fitness to identify *X*_*Gbest*_, *X*_*Lbest*_.

  **while** *t*<*T* **do**

   Calculate the temperature using Eq 3

   **if** temperature > 30 **then**

    Compute *X*_*shade*_ via Eq 4

    **if** rand < 0.5 **then**

     Adjust the location by using Eq 5

    **else**

     Adjust the location by using Eq 24

    **end if**

   **else**

    Evaluate the relationship between food intake proportion and food size using Eqs 9 and 11

    **if** Q > (C3 + 1)/2 **then**

     Adjust the location by using Eq 13

    **else**

     Generate improved Lévy Flight random numbers via Eqs 15 and 16

     Adjust the location by using Eq 17

    **end if**

   **end if**

   **for** 1:N **do**

    Adjust fitness values, *X*_*Gbest*_, *X*_*Lbest*_

   **end for**

   *t* = *t* + 1

  **end while**

### 3.5 Computational time complexity

To better evaluate the performance of each algorithm, this paper employs a method where each assessment of the fitness function counts as one iteration. This is because some algorithms perform numerous position evaluations during iteration; using this approach allows for a more accurate comparison of the algorithms’ performance differences.In the subsequent test set, the iteration count is indirectly determined by the problem’s dimensionality. Here, T denotes the result of multiplying the problem’s dimensionality by a predetermined multiplier. Different algorithms evaluate the fitness function a varying number of times during iteration, potentially precluding direct comparison. For COA, each iteration performs only one fitness analysis, yielding an overall time complexity of O(DxT). The improved strategy during iterations does not invoke the fitness function when computing Euclidean distances. Whilst this introduces additional runtime overhead, it does not impact the overall time complexity. Similarly, the Levy flight strategy imposes no time overhead related to D, N, or T, thus also preserving the overall time complexity.The CPU time statistics for multiple dimensions from CEC2020 reveal that as the number of dimensions increases, the impact of these additional overheads on algorithmic time diminishes progressively. This finding corroborates the preceding theoretical analysis.

In terms of overall computational time, comparative experiments were conducted on the CEC-2020 benchmark under three-dimensional settings (20D, 30D, and 50D). All algorithms are evaluated using identical parameter configurations and executed within the same computational environment to ensure a fair comparison. The results show that, as dimensionality increases, the runtime of ICOA remains within a reasonable range, demonstrating good scalability and computational stability. Furthermore, compared to other benchmark algorithms, ICOA displays a slower growth in computational cost at higher dimensions, indicating superior computational efficiency. The overall runtime comparison of ICOA across different dimensions of the CEC2020 benchmark functions is presented in the Table 1.

The comparison of time complexity between COA and ICOA is presented in Table 2.

**Table 2:** Comparison of computational complexity: COA and ICOA.

Phase	COA Complexity	ICOA Complexity	Description
Initialization	O(D×N)	O(D×N)	Both algorithms generate initial positions for *N* population in a *D*-dimensional space. ICOA uses Sobol sequences, but complexity remains unchanged.
Iteration	O(D×T)	O(D×T)	COA evaluates the entire population each iteration. ICOA reduces dependency on *N* by limiting fitness evaluations.
Total Complexity	O(D×T)+O(D×N)	O(D×N)+O(D×T)	For large *N*, COA has higher computational cost. ICOA optimizes the iteration phase for better efficiency.
**Notation:** Population *N*, Iteration *T*, Dimension *D*.			

## 4 Experimental results and discussion

This section presents simulation experiments to evaluate the optimization performance and effectiveness of the ICOA.

This study assesses the ICOA’s ability to optimize diverse objective functions using the CEC-2019 benchmark suite and CEC-2020 test set. The results are benchmarked against established algorithms, including COA, SCA, WOA, HHO, WDO, PSO, APO, DBO, and BKA, to thoroughly evaluate ICOA’s solution accuracy and optimization efficacy. Four key metrics—mean value (Mean), standard deviation (Std), best-found value(Best), and rank value(Rank)—are utilized for a comprehensive performance analysis.

### 4.1 Parameter setting and environment

The hardware and software configurations for all experiments are detailed in Table 3. The experimental parameters include the dimension of the population (D=20, 30, 50), while keeping the population size of N=100 and a fixed number of iterations (MaxFEs=10000). For each algorithm, 30 independent runs are performed, and statistical metrics (Mean, Std, Best, and Rank) are collected. The details of the parameters for each algorithm are presented in Table 4.

**Table 3:** Hardware and software used in all experiments.

Item	Hardware/Software	Description
Hardware	Hard Drive	1000GB
	RAM	192GB
	CPU	Intel XEON 8175M
Software	Language	MATLAB 2025b
	Operating System	Windows Serve 2022

**Table 4:** Algorithm parameter configurations.

Algorithm	Parameter Setting	Value
ICOA	*C*_3_	3
	*μ*	25
	*σ*	3
COA [34]	*C*_3_	3
	*μ*	25
	*σ*	3
SCA [19]	*α*	2
HHO [31]	*β*	1.5
	*μ*	(0,1)
	*v*	(0,1)
WOA [30]	*a*	2 → 0
	*b*	1
	$r_{1},r_{2},p$	(0,1)
WDO [28]	*RT*	3
	*g*	0.2
	*alp*	0.4
	*c*	0.4
PSO [32]	*C*_1_	2
	*C*_2_	2
APO [63]	p/ω	0.2
	*β*	(0.01,0.1)
	*α*	(0.8,1.2)
DBO [64]	P_percent	0.2
BKA [65]	*p*	0.9

### 4.2 Ensitivity analysis

To evaluate the stability and robustness of the proposed Improved Crayfish Optimization Algorithm (ICOA) under varying parameter configurations, a systematic parameter sensitivity analysis is conducted in this section. Internal parameter settings significantly influence algorithmic performance; appropriate tuning not only enhances global search capability and convergence speed but also strengthens generalization across complex optimization problems. Given that ICOA incorporates stochastic factors and the Lévy flight mechanism in population updating and step-size control, two core parameter categories are selected for analysis: one is the random weight parameter rand, which modulates perturbation intensity in individual search; and the other is the Lévy flight distribution parameter β, which governs the heavy-tailed step-length distribution and exploration range.

Experiments are performed on the CEC-2020 benchmark suite at 30 dimensions, with systematic combinations of rand∈{0.3,0.5,0.8} and β∈{0.5,0.8,1.2,1.8}. Table 5 is conducted on the CEC-2020 benchmark suite at 30 dimensions, to ensure statistical reliability and reproducibility, a population size of 100 is adopted, and each parameter configuration is independently executed for 10000 runs. Performance is evaluated using mean fitness (Mean), standard deviation (Std), and best-found value (Best), providing a comprehensive assessment of convergence precision, stability, and global search ability. Results reveal moderate sensitivity to *rand*, in contrast to relative insensitivity to *β*. Notably, the configuration rand=0.3 and β≈0.8 achieves an optimal trade-off between convergence precision and stability. These findings provide a data-driven foundation for parameter selection and facilitate broader deployment of ICOA in real-world optimization scenarios.

**Table 5:** The influence of parameter *rand* and *β* on test results (CEC-2020).

Functions	Statistics	rand=0.3,*β* = 0.8	rand=0.5,*β*=0.8	rand=0.8,*β*=0.8	r=0.3,*β*=0.5	r=0.3,*β*=1.2	r=0.3,*β*=1.8
F1	Mean	**8.15× 10** ^**3**^	**9.99× 10** ^**3**^	**8.88× 10** ^**3**^	**2.38× 10** ^**4**^	**1.09× 10** ^**5**^	**1.08× 10** ^**4**^
	Std	**5.58× 10** ^**3**^	**1.09× 10** ^**4**^	**8.38× 10** ^**3**^	**8.19× 10** ^**4**^	**4.33× 10** ^**5**^	**1.05× 10** ^**4**^
	Best	6.10× 10^2^	6.38× 10^2^	**4.80× 10** ^**2**^	**6.10× 10** ^**2**^	**1.52× 10** ^**3**^	**7.68× 10** ^**2**^
F2	Mean	**4.94× 10** ^**3**^	**5.28× 10** ^**3**^	**5.12× 10** ^**3**^	**4.99× 10** ^**3**^	**5.15× 10** ^**3**^	**5.21× 10** ^**3**^
	Std	7.49× 10^2^	8.14× 10^2^	7.08× 10^2^	7.49× 10^2^	**5.02× 10** ^**2**^	**8.07× 10** ^**2**^
	Best	**3.53× 10** ^**3**^	**3.65× 10** ^**3**^	**3.74× 10** ^**3**^	**3.55× 10** ^**3**^	**3.92× 10** ^**3**^	**3.75× 10** ^**3**^
F3	Mean	**9.19× 10** ^**2**^	**9.70× 10** ^**2**^	**9.59× 10** ^**2**^	**9.82× 10** ^**2**^	**9.83× 10** ^**2**^	**1.02× 10** ^**3**^
	Std	**9.04$\times 10^{1}$**	**1.28× 10** ^**2**^	**1.66× 10** ^**2**^	**1.13× 10** ^**2**^	**1.15× 10** ^**2**^	**1.12× 10** ^**2**^
	Best	**7.85× 10** ^**2**^	**7.96× 10** ^**2**^	**7.86× 10** ^**2**^	**8.13× 10** ^**2**^	**8.30× 10** ^**2**^	**8.42× 10** ^**2**^
F4	Mean	**1.90× 10** ^**3**^	**1.90× 10** ^**3**^	**1.90× 10** ^**3**^	**1.90× 10** ^**3**^	**1.90× 10** ^**3**^	**1.90× 10** ^**3**^
	Std	**0.00**	**0.00**	**0.00**	**0.00**	**0.00**	**0.00**
	Best	**1.90× 10** ^**3**^	**1.90× 10** ^**3**^	**1.90× 10** ^**3**^	**1.90× 10** ^**3**^	**1.90× 10** ^**3**^	**1.90× 10** ^**3**^
F5	Mean	**2.80× 10** ^**4**^	**3.71× 10** ^**4**^	**3.07× 10** ^**4**^	**3.40× 10** ^**4**^	**3.46× 10** ^**4**^	**4.10× 10** ^**4**^
	Std	1.49× 10^4^	1.92× 10^4^	**1.29× 10** ^**4**^	**1.88× 10** ^**4**^	**1.58× 10** ^**4**^	**2.38× 10** ^**4**^
	Best	9.68× 10^3^	1.49× 10^4^	1.59× 10^4^	**9.41× 10** ^**3**^	**1.16× 10** ^**4**^	**1.21× 10** ^**4**^
F6	Mean	**1.91× 10** ^**3**^	**1.95× 10** ^**3**^	**1.92× 10** ^**3**^	**1.95× 10** ^**3**^	**2.07× 10** ^**3**^	**2.06× 10** ^**3**^
	Std	**1.11× 10** ^**2**^	**1.30× 10** ^**2**^	**1.20× 10** ^**2**^	**1.25× 10** ^**2**^	**1.77× 10** ^**2**^	**1.82× 10** ^**2**^
	Best	1.68× 10^3^	1.73× 10^3^	**1.67× 10** ^**3**^	**1.75× 10** ^**3**^	**1.79× 10** ^**3**^	**1.74× 10** ^**3**^
F7	Mean	1.64× 10^4^	1.29× 10^4^	**9.62× 10** ^**3**^	**1.64× 10** ^**4**^	**1.61× 10** ^**4**^	**1.85× 10** ^**4**^
	Std	4.46× 10^3^	2.98× 10^3^	**1.90× 10** ^**3**^	**5.41× 10** ^**3**^	**4.88× 10** ^**3**^	**8.40× 10** ^**3**^
	Best	8.73× 10^3^	7.37× 10^3^	**7.01× 10** ^**3**^	**8.13× 10** ^**3**^	**7.62× 10** ^**3**^	**7.71× 10** ^**3**^
F8	Mean	**2.30× 10** ^**3**^	**2.31× 10** ^**3**^	**2.59× 10** ^**3**^	**2.30× 10** ^**3**^	**2.30× 10** ^**3**^	**2.30× 10** ^**3**^
	Std	**1.84**	**3.57$\times 10^{1}$**	**1.11× 10** ^**3**^	**3.56**	**2.63**	**3.16**
	Best	**2.30× 10** ^**3**^	**2.30× 10** ^**3**^	**2.30× 10** ^**3**^	**2.30× 10** ^**3**^	**2.30× 10** ^**3**^	**2.30× 10** ^**3**^
F9	Mean	**2.93× 10** ^**3**^	**2.95× 10** ^**3**^	**2.97× 10** ^**3**^	**2.98× 10** ^**3**^	**2.94× 10** ^**3**^	**2.94× 10** ^**3**^
	Std	**3.36$\times 10^{1}$**	**3.53$\times 10^{1}$**	**7.47$\times 10^{1}$**	**6.57$\times 10^{1}$**	**3.79$\times 10^{1}$**	**5.35$\times 10^{1}$**
	Best	2.88× 10^3^	2.89× 10^3^	2.88× 10^3^	2.89× 10^3^	**2.86× 10** ^**3**^	**2.88× 10** ^**3**^
F10	Mean	**2.90× 10** ^**3**^	**2.90× 10** ^**3**^	**2.91× 10** ^**3**^	**2.91× 10** ^**3**^	**2.90× 10** ^**3**^	**2.90× 10** ^**3**^
	Std	2.03$\times 10^{1}$	**1.95$\times 10^{1}$**	**1.98$\times 10^{1}$**	**2.48$\times 10^{1}$**	**2.11$\times 10^{1}$**	**2.07$\times 10^{1}$**
	Best	**2.88× 10** ^**3**^	**2.88× 10** ^**3**^	**2.88× 10** ^**3**^	**2.88× 10** ^**3**^	**2.88× 10** ^**3**^	**2.88× 10** ^**3**^

### 4.3 CEC2019 benchmark test function experiment

#### 4.3.1 Numerical experiment and analysis.

This part aims to comparatively assess the performance of ICOA and other algorithms (including COA, SCA, WOA, HHO, WDO, PSO, APO, DBO and BKA), to evaluate their optimization capabilities.

In CEC2019, except for the function $F_{1}(d=9),F_{2}(d=16),F_{3}(d=18)$, the functions all have the same dimensions (d=10) and the same search intervals. Here, ‘Mean’ denotes the average value of the objective function, reflecting the algorithm’s computational accuracy. ‘Std’ represents the standard deviation of the best objective function values, indicating the algorithm’s stability. ‘Best’ refers to the optimal value obtained, while ‘Rank’ shows the ranking based on the best results.

Table 6 shows that ICOA performs significantly better precision in obtaining optimal values for functions. Specifically, the ‘Best’ and the ‘Mean’ of ICOA is significantly better than that of other algorithms, ‘Std’ of ICOA is slightly less than the ‘Best’. This shows that ICOA has good optimization ability. In contrast to the initialization and updating methods used in the traditional COA, the ICOA promotes greater information exchange among individuals. This enhancement strengthens the search and optimization capabilities of the algorithm.

**Table 6:** Experimental outcomes for each algorithm IEEE2019 (optimal data are highlighted in bold).

Functions	Statistics	ICOA	COA	SCA	WOA	HHO	WDO	PSO	APO	DBO	BKA
F1	Mean	**1.00**	**1.00**	**8.46× 10** ^**5**^	**3.58× 10** ^**6**^	**1.00**	**1.73$\times 10^{1}$**	**3.69× 10** ^**8**^	**1.00**	**2.50× 10** ^**6**^	**8.89× 10** ^**7**^
	Std	**0.00**	**0.00**	**2.49× 10** ^**6**^	**6.51× 10** ^**6**^	**0.00**	**5.04$\times 10^{1}$**	**1.98× 10** ^**8**^	**0.00**	**3.92× 10** ^**6**^	**3.72× 10** ^**7**^
	Best	**1.00**	**1.00**	**1.00**	**6.68× 10** ^**2**^	**1.00**	**1.00**	**6.23× 10** ^**7**^	**1.00**	**2.70× 10** ^**3**^	**1.40× 10** ^**7**^
	Rank	**1**	**1**	6	7	**1**	**1**	10	**1**	8	9
F2	Mean	**4.42**	**4.67**	**1.66× 10** ^**3**^	**7.60× 10** ^**3**^	**4.97**	**4.43**	**1.97× 10** ^**4**^	**4.59**	**9.41× 10** ^**2**^	**9.17× 10** ^**3**^
	Std	3.91E-01	3.83E-01	9.84× 10^2^	2.85× 10^3^	**7.26E-02**	**1.07E-01**	**5.12× 10** ^**3**^	**4.24E-01**	**1.25× 10** ^**3**^	**1.24× 10** ^**3**^
	Best	4.03	4.05	1.36× 10^2^	2.14× 10^3^	4.70	**4.01**	**1.08× 10** ^**4**^	**4.03**	**2.53× 10** ^**2**^	**6.85× 10** ^**3**^
	Rank	3	4	6	8	5	**1**	10	2	7	9
F3	Mean	2.21	4.53	7.56	3.50	4.23	1.52	7.90	**1.33**	**6.81**	**7.49**
	Std	1.59	2.49	1.70	2.07	9.55E-01	3.47E-01	8.57E-01	**1.66E-01**	**1.74**	**9.74E-01**
	Best	**1.00**	**1.41**	**4.93**	**1.41**	**2.63**	**1.01**	**6.64**	**1.00**	**1.41**	**5.54**
	Rank	1	5	8	6	7	**3**	10	2	7	10
F4	Mean	**2.20$\times 10^{1}$**	**2.76$\times 10^{1}$**	**4.03$\times 10^{1}$**	**4.42$\times 10^{1}$**	**5.81$\times 10^{1}$**	**4.08$\times 10^{1}$**	**3.66$\times 10^{1}$**	**2.44$\times 10^{1}$**	**2.48$\times 10^{1}$**	**7.79$\times 10^{1}$**
	Std	1.72$\times 10^{1}$	1.68$\times 10^{1}$	**6.61**	**1.52$\times 10^{1}$**	**1.48$\times 10^{1}$**	**1.49$\times 10^{1}$**	**9.73**	**1.33$\times 10^{1}$**	**9.62**	**6.94**
	Best	6.10	**4.98**	**3.12$\times 10^{1}$**	**2.09$\times 10^{1}$**	**2.70$\times 10^{1}$**	**1.39$\times 10^{1}$**	**1.73$\times 10^{1}$**	**5.98**	**6.97**	**5.70$\times 10^{1}$**
	Rank	3	**1**	9	7	8	5	6	2	4	10
F5	Mean	**1.10**	**1.13**	**6.21**	**1.76**	**3.56$\times 10^{1}$**	**2.29**	**1.57**	**1.80**	**1.14**	**4.26$\times 10^{1}$**
	Std	**4.73E-02**	**9.60E-02**	**1.74**	**3.53E-01**	**2.21$\times 10^{1}$**	**3.14E-01**	**7.01E-02**	**7.63E-01**	**7.90E-02**	**1.07$\times 10^{1}$**
	Best	1.03	**1.00**	**3.58**	**1.26**	**4.31**	**1.97**	**1.40**	**1.03**	**1.04**	**1.99$\times 10^{1}$**
	Rank	2	**1**	8	5	9	7	6	3	4	10
F6	Mean	3.52	3.34	6.97	7.79	8.90	4.31	4.76	**2.72**	**4.95**	**1.05$\times 10^{1}$**
	Std	1.18	1.52	1.09	1.76	1.72	1.49	1.43	8.57E-01	2.42	**7.81E-01**
	Best	1.04	1.03	5.61	5.00	5.58	2.21	1.83	1.14	**1.01**	**8.43**
	Rank	3	2	9	7	8	6	5	4	**1**	10
F7	Mean	**7.80× 10** ^**2**^	**8.71× 10** ^**2**^	**1.26× 10** ^**3**^	**1.19× 10** ^**3**^	**1.31× 10** ^**3**^	**1.30× 10** ^**3**^	**1.13× 10** ^**3**^	**9.07× 10** ^**2**^	**8.23× 10** ^**2**^	**1.63× 10** ^**3**^
	Std	3.49× 10^2^	2.85× 10^2^	2.14× 10^2^	3.17× 10^2^	2.76× 10^2^	3.34× 10^2^	2.80× 10^2^	4.41× 10^2^	3.45× 10^2^	**1.59× 10** ^**2**^
	Best	**1.56× 10** ^**2**^	**2.53× 10** ^**2**^	**8.26× 10** ^**2**^	**6.26× 10** ^**2**^	**8.28× 10** ^**2**^	**7.04× 10** ^**2**^	**4.92× 10** ^**2**^	**2.42× 10** ^**2**^	**2.42× 10** ^**2**^	**1.29× 10** ^**3**^
	Rank	**1**	4	8	6	9	7	5	3	2	10
F8	Mean	**3.54**	**3.74**	**4.17**	**4.51**	**4.58**	**4.27**	**3.98**	**4.79**	**3.95**	**4.66**
	Std	3.62E-01	3.87E-01	2.72E-01	2.84E-01	2.78E-01	3.96E-01	3.28E-01	2.99E-01	4.64E-01	**1.60E-01**
	Best	**2.81**	**2.82**	**3.66**	**3.82**	**4.14**	**3.67**	**3.48**	**3.90**	**2.82**	**4.24**
	Rank	**1**	2	5	7	9	6	4	8	3	10
F9	Mean	1.18	1.22	1.46	1.35	1.94	1.33	1.23	**1.09**	**1.28**	**3.15**
	Std	5.27E-02	8.37E-02	1.03E-01	1.53E-01	6.64E-01	1.28E-01	9.06E-02	**4.63E-02**	**1.39E-01**	**3.19E-01**
	Best	1.09	1.10	1.27	1.12	1.19	1.13	1.10	**1.03**	**1.08**	**2.52**
	Rank	3	5	9	6	8	7	4	**1**	2	10
F10	Mean	**1.54$\times 10^{1}$**	**1.86$\times 10^{1}$**	**2.14$\times 10^{1}$**	**2.08$\times 10^{1}$**	**2.11$\times 10^{1}$**	**2.09$\times 10^{1}$**	**2.14$\times 10^{1}$**	**1.99$\times 10^{1}$**	**2.12$\times 10^{1}$**	**2.14$\times 10^{1}$**
	Std	8.31	6.16	8.50E-02	1.38	1.38E-01	2.04	7.40E-02	4.24	1.26E-01	**6.86E-02**
	Best	**1.00**	**2.28**	**2.12$\times 10^{1}$**	**1.36$\times 10^{1}$**	**2.10$\times 10^{1}$**	**1.01$\times 10^{1}$**	**2.12$\times 10^{1}$**	**3.20**	**2.10$\times 10^{1}$**	**2.12$\times 10^{1}$**
	Rank	**1**	2	8	5	6	4	9	3	7	10
Average	Mean	**8.35$\times 10^{1}$**	**9.37$\times 10^{1}$**	**8.49× 10** ^**4**^	**3.59× 10** ^**5**^	**1.45× 10** ^**2**^	**1.40× 10** ^**2**^	**3.69× 10** ^**7**^	**9.69$\times 10^{1}$**	**2.50× 10** ^**5**^	**8.89× 10** ^**6**^
	Std	3.78$\times 10^{1}$	**3.13$\times 10^{1}$**	**2.50× 10** ^**5**^	**6.51× 10** ^**5**^	**3.17$\times 10^{1}$**	**4.04$\times 10^{1}$**	**1.99× 10** ^**7**^	**4.61$\times 10^{1}$**	**3.92× 10** ^**5**^	**3.72× 10** ^**6**^
	Best	**1.75$\times 10^{1}$**	**2.73$\times 10^{1}$**	**1.03× 10** ^**2**^	**3.48× 10** ^**2**^	**8.99$\times 10^{1}$**	**7.43$\times 10^{1}$**	**6.23× 10** ^**6**^	**2.64$\times 10^{1}$**	**3.23× 10** ^**2**^	**1.40× 10** ^**6**^
	Rank	**1**	3	6	8	5	4	10	2	7	9

#### 4.3.2 Iterative curve analysis.

**Fig 4:**
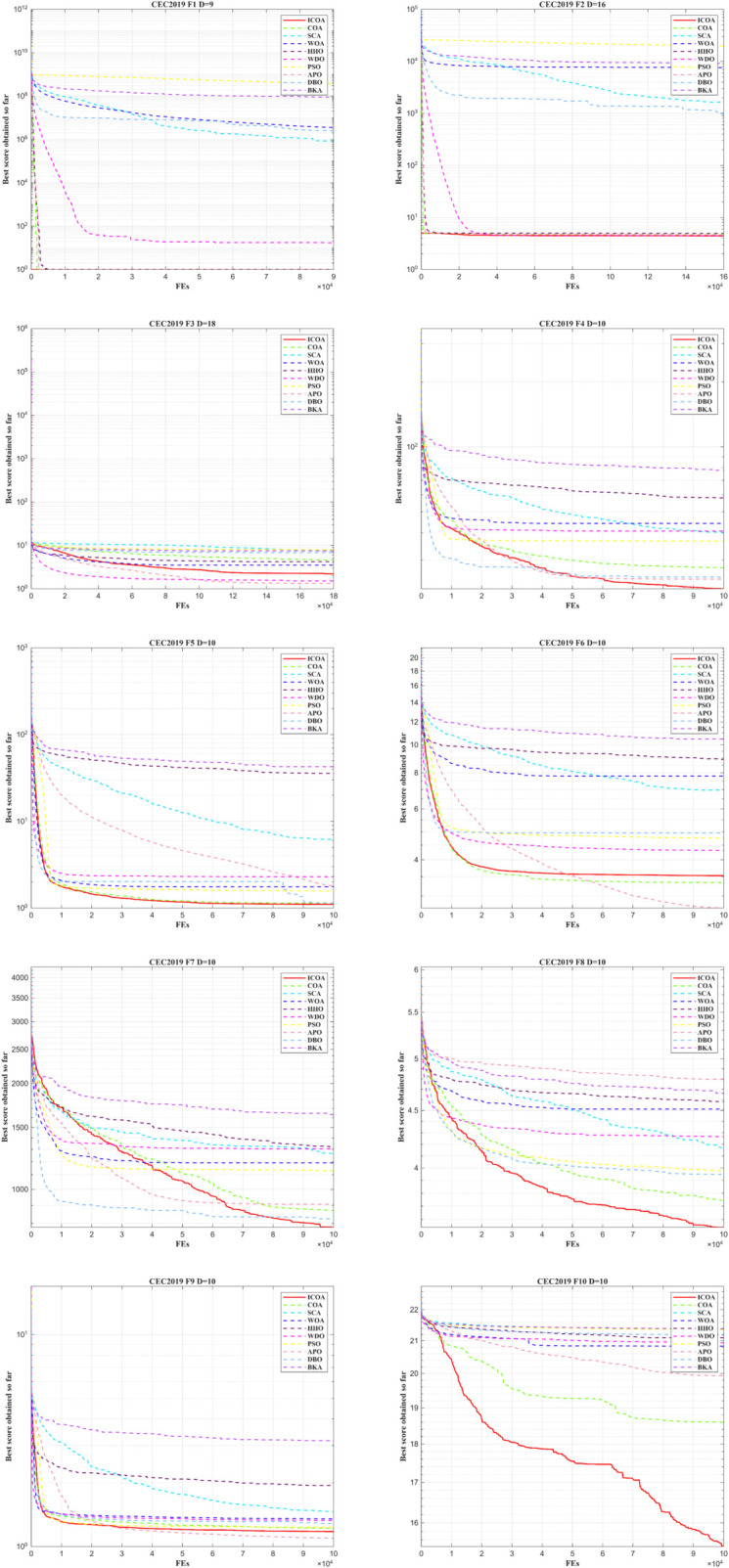
Convergence curve of ICOA in IEEE CEC2019 (DIM=10).

To more effectively examine the convergence of the ICOA in CEC2019, the convergence curve is presented in IEEE CEC2019 in Fig 4. The x-axis shows how many iterations were performed, and the y-axis shows the average result over 30 independent runs.

Firstly, during the early phase of iteration, the convergence curve of ICOA decreases rapidly. This suggests that initializing data with the *Sobol* sequence effectively accelerates convergence. Furthermore, ICOA shows strong global search ability and seldom falls into local optima throughout iterations. In $F_{7},F_{8}$, although early convergence is slightly slower, other algorithms generally converge more slowly as the iteration progresses. And the present algorithm can carry out the optimality-seeking process. Incorporating the Lévy flight mode improves its ability to identify potentially optimal solutions. From the vertical comparison, ICOA achieves more effective performance outcomes, particularly showing rapid convergence. This improvement in optimization accuracy is mainly due to the Euclidean distance-fitness balanced competition strategy, which takes into account the differences between individual positions and fitness values. Enhances the interaction of information between individuals within the population.

**Fig 5:**
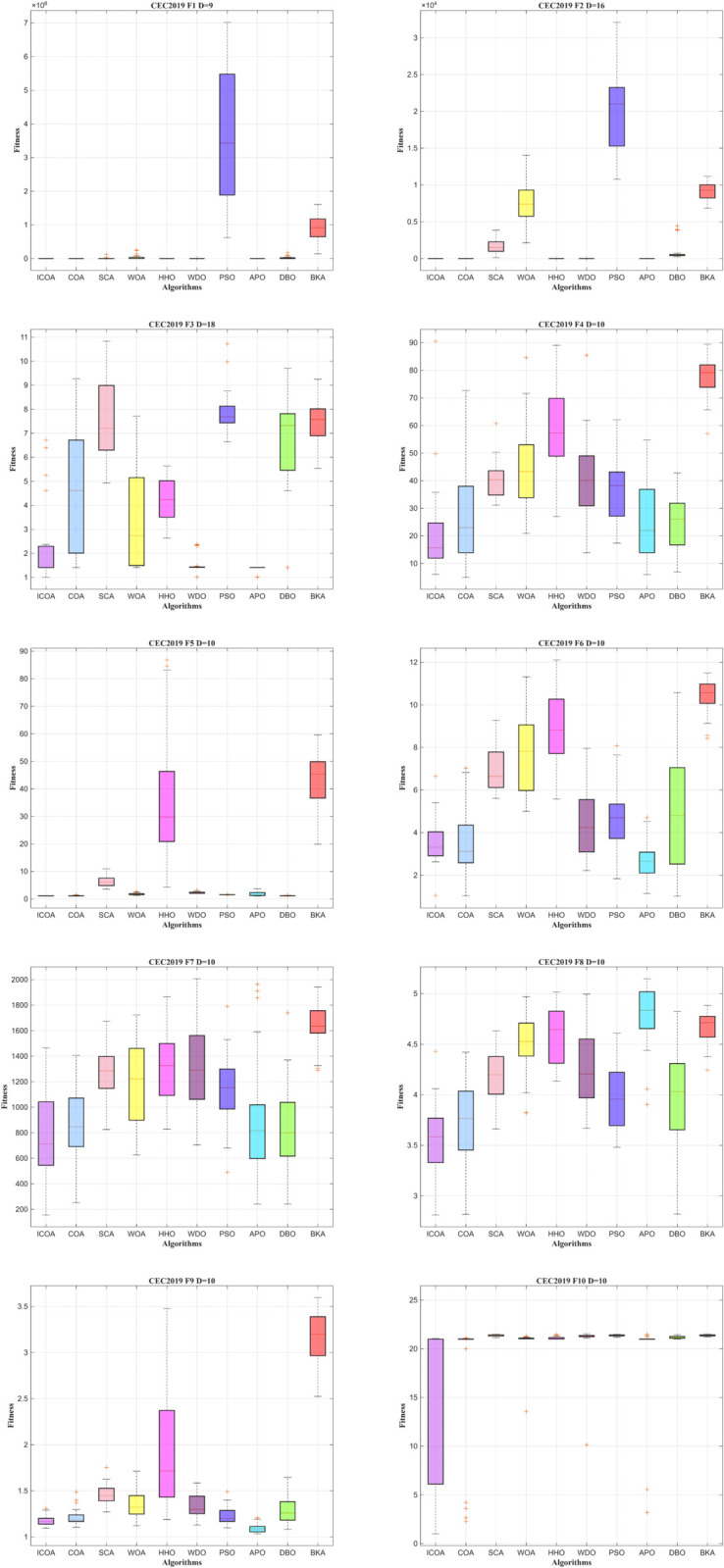
Box plot of ICOA in IEEE CEC2019 (DIM=10).

In Fig 5, detailed box-and-line plots present the performance, clearly indicating that ICOA exhibits superior performance. The solution distribution of ICOA is more concentrated compared to that of all other algorithms. These results showcase ICOA’s strong global search and local refinement abilities, confirming its reliability and precision.

### 4.4 CEC2020(DIM=20) benchmark test function experiment

Table 7 shows that the ICOA algorithm performs exceptionally well overall in terms of average fitness (Mean) on the CEC2020 test function set (dimension D=20). It can be observed that ICOA achieves lower mean fitness values in most test functions compared with COA, SCA, WOA, HHO, WDO, PSO, APO, DBO, and BKA, demonstrating its superior global search capability and convergence stability. Specifically, for unimodal functions (e.g., F1 and F2), ICOA achieves significantly lower mean fitness values, indicating faster and more accurate convergence. On multimodal and hybrid functions (F6–F9), ICOA still maintains competitive performance with lower mean values, suggesting it has a stronger ability to avoid local optima. On certain high-dimensional or discontinuous functions (e.g., F10), ICOA performs comparably to the best competitors and shows good robustness. The overall average ranking also indicates that ICOA ranks among the top algorithms across all functions, confirming its overall optimization capability. In summary, the ICOA algorithm achieves a good balance between global exploration and local exploitation, with fast convergence speed and high stability in fitness, demonstrating strong generalization ability across different types of target functions.

**Table 7:** Experimental outcomes for each algorithm in IEEE2020 D=20 (optimal data are highlighted in bold).

Functions	Statistics	ICOA	COA	SCA	WOA	HHO	WDO	PSO	APO	DBO	BKA
F1	Mean	**2.43× 10** ^**3**^	**4.30× 10** ^**3**^	**5.06× 10** ^**9**^	**2.93× 10** ^**5**^	**1.78× 10** ^**10**^	**1.29× 10** ^**7**^	**4.43× 10** ^**6**^	**1.42× 10** ^**9**^	**1.05× 10** ^**4**^	**2.29× 10** ^**10**^
	Std	**3.12× 10** ^**3**^	**3.71× 10** ^**3**^	**1.23× 10** ^**9**^	**1.29× 10** ^**5**^	**6.05× 10** ^**9**^	**1.16× 10** ^**7**^	**6.99× 10** ^**5**^	**1.09× 10** ^**9**^	**9.37× 10** ^**3**^	**3.39× 10** ^**9**^
	Best	2.32× 10^2^	1.59× 10^2^	2.98× 10^9^	1.02× 10^5^	7.70× 10^9^	4.92× 10^6^	2.72× 10^6^	9.79× 10^7^	**1.01× 10** ^**2**^	**1.59× 10** ^**10**^
	Rank	3	2	8	4	9	6	5	7	**1**	10
F2	Mean	**2.51× 10** ^**3**^	**2.95× 10** ^**3**^	**4.75× 10** ^**3**^	**3.58× 10** ^**3**^	**4.50× 10** ^**3**^	**4.02× 10** ^**3**^	**3.27× 10** ^**3**^	**3.16× 10** ^**3**^	**3.04× 10** ^**3**^	**5.13× 10** ^**3**^
	Std	6.56× 10^2^	8.10× 10^2^	3.26× 10^2^	5.17× 10^2^	4.33× 10^2^	5.28× 10^2^	5.37× 10^2^	5.26× 10^2^	5.88× 10^2^	**2.84× 10** ^**2**^
	Best	1.60× 10^3^	**1.25× 10** ^**3**^	**4.17× 10** ^**3**^	**2.77× 10** ^**3**^	**3.68× 10** ^**3**^	**3.12× 10** ^**3**^	**1.82× 10** ^**3**^	**2.27× 10** ^**3**^	**1.80× 10** ^**3**^	**4.51× 10** ^**3**^
	Rank	2	**1**	9	6	8	7	4	5	3	10
F3	Mean	**7.88× 10** ^**2**^	**8.67× 10** ^**2**^	**9.02× 10** ^**2**^	**9.23× 10** ^**2**^	**9.65× 10** ^**2**^	**8.21× 10** ^**2**^	**8.26× 10** ^**2**^	**8.32× 10** ^**2**^	**7.97× 10** ^**2**^	**1.37× 10** ^**3**^
	Std	3.05$\times 10^{1}$	5.87$\times 10^{1}$	**1.93$\times 10^{1}$**	**4.88$\times 10^{1}$**	**3.50$\times 10^{1}$**	**2.29$\times 10^{1}$**	**2.36$\times 10^{1}$**	**3.61$\times 10^{1}$**	**7.24$\times 10^{1}$**	**7.45$\times 10^{1}$**
	Best	7.41× 10^2^	7.42× 10^2^	8.58× 10^2^	8.24× 10^2^	8.94× 10^2^	7.85× 10^2^	7.86× 10^2^	7.82× 10^2^	**7.31× 10** ^**2**^	**1.24× 10** ^**3**^
	Rank	2	3	8	7	9	5	6	4	**1**	10
F4	Mean	**1.90× 10** ^**3**^	**1.90× 10** ^**3**^	**1.90× 10** ^**3**^	**1.90× 10** ^**3**^	**1.90× 10** ^**3**^	**1.90× 10** ^**3**^	**1.91× 10** ^**3**^	**1.90× 10** ^**3**^	**1.90× 10** ^**3**^	**2.23× 10** ^**3**^
	Std	**0.00**	**0.00**	**1.83**	**5.72E-01**	**0.00**	**2.20**	**1.24**	**0.00**	**1.64**	**2.82× 10** ^**2**^
	Best	**1.90× 10** ^**3**^	**1.90× 10** ^**3**^	**1.90× 10** ^**3**^	**1.90× 10** ^**3**^	**1.90× 10** ^**3**^	**1.90× 10** ^**3**^	**1.90× 10** ^**3**^	**1.90× 10** ^**3**^	**1.90× 10** ^**3**^	**1.93× 10** ^**3**^
	Rank	**1**	**1**	**1**	**1**	**1**	**1**	9	**1**	8	10
F5	Mean	**1.02× 10** ^**4**^	**1.18× 10** ^**5**^	**1.08× 10** ^**6**^	**8.54× 10** ^**5**^	**3.26× 10** ^**6**^	**7.22× 10** ^**4**^	**1.06× 10** ^**5**^	**1.29× 10** ^**4**^	**3.49× 10** ^**5**^	**3.64× 10** ^**6**^
	Std	**2.77× 10** ^**3**^	**6.42× 10** ^**4**^	**7.03× 10** ^**5**^	**9.34× 10** ^**5**^	**3.14× 10** ^**6**^	**2.83× 10** ^**4**^	**5.66× 10** ^**4**^	**4.51× 10** ^**4**^	**5.18× 10** ^**5**^	**1.37× 10** ^**6**^
	Best	5.15× 10^3^	1.26× 10^4^	2.70× 10^5^	2.83× 10^4^	3.24× 10^5^	1.40× 10^4^	1.72× 10^4^	**2.02× 10** ^**3**^	**1.08× 10** ^**4**^	**1.45× 10** ^**6**^
	Rank	2	4	8	7	9	5	6	**1**	3	10
F6	Mean	**1.74× 10** ^**3**^	**1.80× 10** ^**3**^	**2.18× 10** ^**3**^	**2.12× 10** ^**3**^	**2.47× 10** ^**3**^	**2.27× 10** ^**3**^	**2.10× 10** ^**3**^	**1.88× 10** ^**3**^	**1.93× 10** ^**3**^	**2.99× 10** ^**3**^
	Std	**1.12× 10** ^**2**^	**1.70× 10** ^**2**^	**1.86× 10** ^**2**^	**2.46× 10** ^**2**^	**2.68× 10** ^**2**^	**2.28× 10** ^**2**^	**2.10× 10** ^**2**^	**1.51× 10** ^**2**^	**1.70× 10** ^**2**^	**1.64× 10** ^**2**^
	Best	**1.60× 10** ^**3**^	**1.61× 10** ^**3**^	**1.88× 10** ^**3**^	**1.74× 10** ^**3**^	**1.76× 10** ^**3**^	**1.77× 10** ^**3**^	**1.74× 10** ^**3**^	**1.62× 10** ^**3**^	**1.62× 10** ^**3**^	**2.60× 10** ^**3**^
	Rank	**1**	2	9	5	7	8	6	4	3	10
F7	Mean	4.93× 10^3^	1.20× 10^5^	2.98× 10^5^	6.30× 10^5^	1.13× 10^6^	4.01× 10^4^	3.19× 10^4^	**2.42× 10** ^**3**^	**2.18× 10** ^**5**^	**9.45× 10** ^**5**^
	Std	7.83× 10^2^	1.46× 10^5^	2.10× 10^5^	5.84× 10^5^	1.18× 10^6^	2.58× 10^4^	2.25× 10^4^	**1.48× 10** ^**2**^	**2.89× 10** ^**5**^	**4.12× 10** ^**5**^
	Best	3.71× 10^3^	4.66× 10^3^	7.80× 10^4^	5.90× 10^4^	2.99× 10^4^	5.01× 10^3^	5.61× 10^3^	**2.15× 10** ^**3**^	**6.24× 10** ^**3**^	**3.11× 10** ^**5**^
	Rank	2	3	9	8	7	4	5	**1**	6	10
F8	Mean	**2.30× 10** ^**3**^	**2.40× 10** ^**3**^	**3.93× 10** ^**3**^	**3.55× 10** ^**3**^	**5.12× 10** ^**3**^	**2.74× 10** ^**3**^	**3.16× 10** ^**3**^	**2.44× 10** ^**3**^	**3.00× 10** ^**3**^	**4.88× 10** ^**3**^
	Std	**6.71E-01**	**5.30× 10** ^**2**^	**1.80× 10** ^**3**^	**1.67× 10** ^**3**^	**1.19× 10** ^**3**^	**1.08× 10** ^**3**^	**1.46× 10** ^**3**^	**1.30× 10** ^**2**^	**8.67× 10** ^**2**^	**4.43× 10** ^**2**^
	Best	2.30× 10^3^	2.30× 10^3^	2.52× 10^3^	2.31× 10^3^	3.40× 10^3^	2.32× 10^3^	2.31× 10^3^	2.33× 10^3^	**2.30× 10** ^**3**^	**3.89× 10** ^**3**^
	Rank	3	2	8	4	9	6	5	7	**1**	10
F9	Mean	**2.85× 10** ^**3**^	**2.86× 10** ^**3**^	**2.98× 10** ^**3**^	**2.97× 10** ^**3**^	**3.19× 10** ^**3**^	**2.94× 10** ^**3**^	**3.11× 10** ^**3**^	**2.89× 10** ^**3**^	**2.90× 10** ^**3**^	**3.18× 10** ^**3**^
	Std	2.25$\times 10^{1}$	2.69$\times 10^{1}$	**2.12$\times 10^{1}$**	**6.46$\times 10^{1}$**	**1.19× 10** ^**2**^	**5.66$\times 10^{1}$**	**7.05$\times 10^{1}$**	**6.20$\times 10^{1}$**	**3.13$\times 10^{1}$**	**4.78$\times 10^{1}$**
	Best	**2.82× 10** ^**3**^	**2.82× 10** ^**3**^	**2.94× 10** ^**3**^	**2.88× 10** ^**3**^	**3.05× 10** ^**3**^	**2.85× 10** ^**3**^	**2.99× 10** ^**3**^	**2.83× 10** ^**3**^	**2.85× 10** ^**3**^	**3.07× 10** ^**3**^
	Rank	**1**	2	7	6	9	4	8	3	5	10
F10	Mean	2.95× 10^3^	2.96× 10^3^	3.11× 10^3^	3.00× 10^3^	4.18× 10^3^	2.97× 10^3^	2.95× 10^3^	3.01× 10^3^	**2.94× 10** ^**3**^	**4.71× 10** ^**3**^
	Std	3.27$\times 10^{1}$	**3.10$\times 10^{1}$**	**5.46$\times 10^{1}$**	**3.17$\times 10^{1}$**	**6.15× 10** ^**2**^	**3.41$\times 10^{1}$**	**3.33$\times 10^{1}$**	**3.85$\times 10^{1}$**	**3.53$\times 10^{1}$**	**3.10× 10** ^**2**^
	Best	2.91× 10^3^	2.90× 10^3^	3.00× 10^3^	2.92× 10^3^	3.40× 10^3^	2.90× 10^3^	2.90× 10^3^	**2.92× 10** ^**3**^	**2.91× 10** ^**3**^	**4.08× 10** ^**3**^
	Rank	4	2	8	6	9	3	**1**	7	5	10
Average	Mean	**3.26× 10** ^**3**^	**2.58× 10** ^**4**^	**5.06× 10** ^**8**^	**1.79× 10** ^**5**^	**1.78× 10** ^**9**^	**1.31× 10** ^**6**^	**4.58× 10** ^**5**^	**1.42× 10** ^**8**^	**5.93× 10** ^**4**^	**2.29× 10** ^**9**^
	Std	**7.53× 10** ^**2**^	**2.16× 10** ^**4**^	**1.23× 10** ^**8**^	**1.65× 10** ^**5**^	**6.06× 10** ^**8**^	**1.16× 10** ^**6**^	**7.80× 10** ^**4**^	**1.09× 10** ^**8**^	**8.18× 10** ^**4**^	**3.39× 10** ^**8**^
	Best	**2.30× 10** ^**3**^	**3.09× 10** ^**3**^	**2.98× 10** ^**8**^	**2.04× 10** ^**4**^	**7.70× 10** ^**8**^	**4.95× 10** ^**5**^	**2.76× 10** ^**5**^	**9.80× 10** ^**6**^	**3.12× 10** ^**3**^	**1.59× 10** ^**9**^
	Rank	**1**	2	8	4	9	6	5	7	3	10

**Fig 6:**
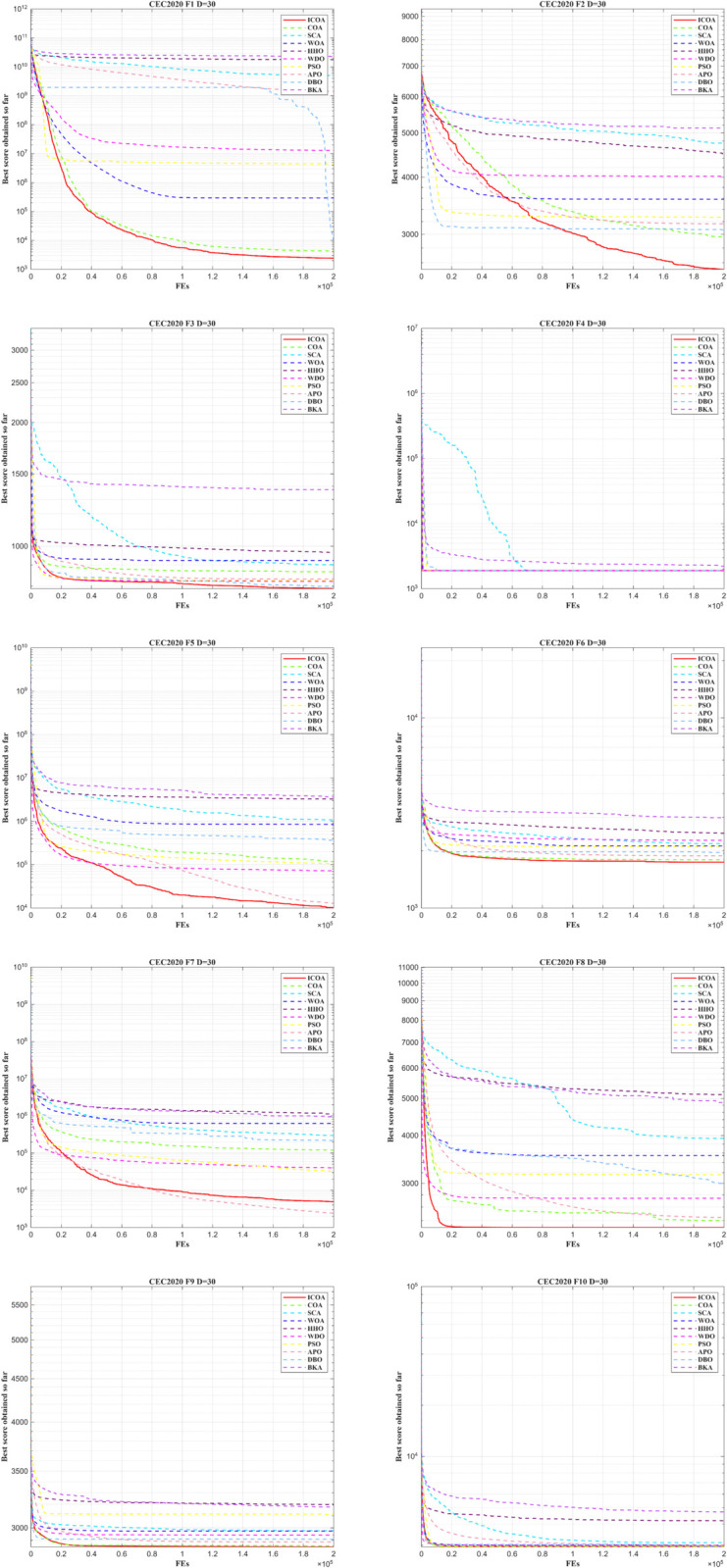
Convergence curve of ICOA in IEEE CEC2020 (DIM=20).

**Fig 7:**
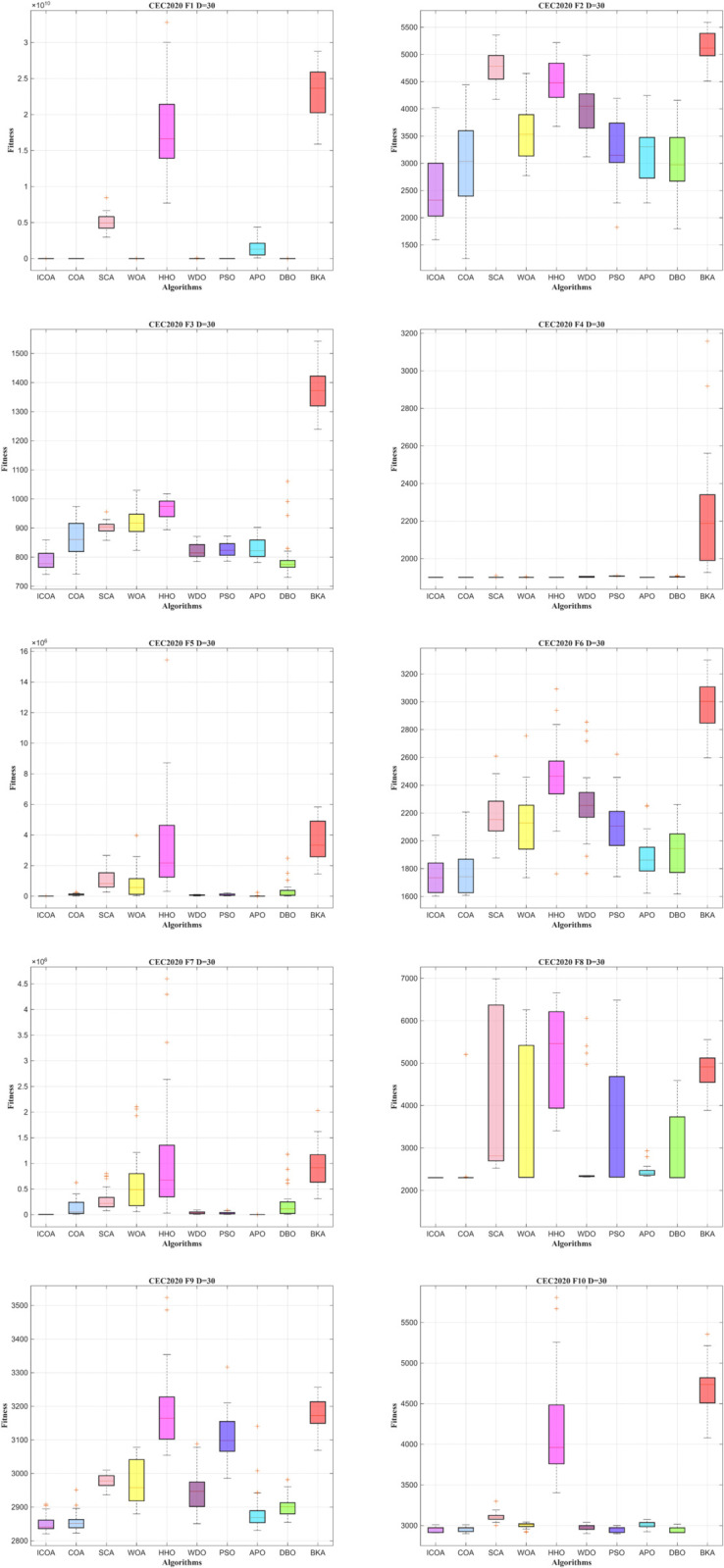
Box plot of ICOA in IEEE CEC2020 (DIM=20).

As illustrated in Fig 6, ICOA exhibits superior convergence accuracy and rapid convergence across all functions except *F*_2_, suggesting its outperformance compared to comparison algorithms. In particular, ICOA demonstrates the highest convergence rate. Furthermore, compared with ten other similar algorithms, ICOA shows more pronounced improvements in terms of optimization capability and prevention of local optima, leading to superior practical applicability. As shown in Fig 7, the detailed box-and-line plots illustrate the performance characteristics, and these visualizations clearly demonstrate that ICOA achieves better performance.

### 4.5 CEC2020(DIM=30) benchmark test function experiment

As shown in Table 8, the results presented in the table demonstrate that, on the CEC2020 benchmark suite (dimension D = 30), the ICOA algorithm achieved the lowest mean fitness values across the majority of test functions. This indicates that ICOA consistently obtains high-quality solutions over multiple independent runs, reflecting its superior global optimization capability and convergence efficiency. Specifically, for most unimodal functions (e.g., F1 and F2), ICOA significantly outperformed the compared algorithms in 30 dimensions, underscoring its clear advantages in overall performance and convergence stability. For complex multimodal and hybrid functions (e.g., F6–F9), ICOA maintained remarkably low mean fitness values even under high-dimensional conditions. Compared with classical algorithms such as PSO, WOA, and HHO, ICOA exhibited stronger global search ability and more effectively escaped local optima. On a few highly discontinuous functions (e.g., F10), although the performance gap narrowed, ICOA still delivered competitive results, demonstrating its robust adaptability.

**Table 8:** Experimental outcomes for each algorithm IEEE2020 D=30 (optimal data are highlighted in bold.

Functions	Statistics	ICOA	COA	SCA	WOA	HHO	WDO	PSO	APO	DBO	BKA
F1	Mean	**1.00× 10** ^**4**^	**1.16× 10** ^**5**^	**1.23× 10** ^**10**^	**2.47× 10** ^**6**^	**3.71× 10** ^**10**^	**9.62× 10** ^**7**^	**1.48× 10** ^**7**^	**2.77× 10** ^**9**^	**1.89× 10** ^**6**^	**5.87× 10** ^**10**^
	Std	**1.29× 10** ^**4**^	**5.41× 10** ^**5**^	**1.73× 10** ^**9**^	**1.73× 10** ^**6**^	**8.31× 10** ^**9**^	**5.56× 10** ^**7**^	**1.70× 10** ^**6**^	**1.24× 10** ^**9**^	**9.18× 10** ^**6**^	**4.73× 10** ^**9**^
	Best	8.49× 10^2^	8.40× 10^2^	9.09× 10^9^	6.11× 10^5^	1.79× 10^10^	1.85× 10^7^	1.06× 10^7^	9.45× 10^8^	**1.02× 10** ^**2**^	**4.91× 10** ^**10**^
	Rank	3	2	8	4	9	6	5	7	**1**	10
F2	Mean	**4.97× 10** ^**3**^	**5.56× 10** ^**3**^	**8.24× 10** ^**3**^	**5.84× 10** ^**3**^	**7.11× 10** ^**3**^	**5.84× 10** ^**3**^	**5.30× 10** ^**3**^	**4.98× 10** ^**3**^	**5.06× 10** ^**3**^	**8.46× 10** ^**3**^
	Std	7.12× 10^2^	7.25× 10^2^	**2.34× 10** ^**2**^	**8.43× 10** ^**2**^	**4.66× 10** ^**2**^	**7.26× 10** ^**2**^	**6.72× 10** ^**2**^	**4.94× 10** ^**2**^	**6.71× 10** ^**2**^	**2.61× 10** ^**2**^
	Best	**3.36× 10** ^**3**^	**3.95× 10** ^**3**^	**7.75× 10** ^**3**^	**4.11× 10** ^**3**^	**6.12× 10** ^**3**^	**4.41× 10** ^**3**^	**4.01× 10** ^**3**^	**4.49× 10** ^**3**^	**3.48× 10** ^**3**^	**7.73× 10** ^**3**^
	Rank	**1**	3	10	5	8	6	4	7	2	9
F3	Mean	**9.27× 10** ^**2**^	**1.12× 10** ^**3**^	**1.13× 10** ^**3**^	**1.19× 10** ^**3**^	**1.32× 10** ^**3**^	**9.74× 10** ^**2**^	**1.07× 10** ^**3**^	**1.01× 10** ^**3**^	**9.47× 10** ^**2**^	**2.02× 10** ^**3**^
	Std	8.75$\times 10^{1}$	1.30× 10^2^	5.24$\times 10^{1}$	7.42$\times 10^{1}$	6.85$\times 10^{1}$	5.47$\times 10^{1}$	**4.12$\times 10^{1}$**	**9.71$\times 10^{1}$**	**7.94$\times 10^{1}$**	**1.54× 10** ^**2**^
	Best	**8.01× 10** ^**2**^	**9.16× 10** ^**2**^	**1.05× 10** ^**3**^	**1.02× 10** ^**3**^	**1.18× 10** ^**3**^	**8.87× 10** ^**2**^	**9.88× 10** ^**2**^	**8.96× 10** ^**2**^	**8.10× 10** ^**2**^	**1.67× 10** ^**3**^
	Rank	**1**	5	8	7	9	3	6	4	2	10
F4	Mean	**1.90× 10** ^**3**^	**1.90× 10** ^**3**^	**1.90× 10** ^**3**^	**1.90× 10** ^**3**^	**1.90× 10** ^**3**^	**1.90× 10** ^**3**^	**1.91× 10** ^**3**^	**1.90× 10** ^**3**^	**1.92× 10** ^**3**^	**2.32× 10** ^**3**^
	Std	**0.00**	**0.00**	**1.70**	**2.92E-02**	**0.00**	**2.94**	**1.84**	**0.00**	**3.67$\times 10^{1}$**	**3.07× 10** ^**2**^
	Best	**1.90× 10** ^**3**^	**1.90× 10** ^**3**^	**1.90× 10** ^**3**^	**1.90× 10** ^**3**^	**1.90× 10** ^**3**^	**1.90× 10** ^**3**^	**1.91× 10** ^**3**^	**1.90× 10** ^**3**^	**1.90× 10** ^**3**^	**1.98× 10** ^**3**^
	Rank	**1**	**1**	**1**	**1**	**1**	**1**	9	**1**	8	10
F5	Mean	**3.08× 10** ^**4**^	**4.63× 10** ^**5**^	**5.12× 10** ^**6**^	**3.86× 10** ^**6**^	**4.35× 10** ^**7**^	**3.05× 10** ^**5**^	**2.20× 10** ^**5**^	**7.60× 10** ^**4**^	**2.02× 10** ^**6**^	**2.41× 10** ^**7**^
	Std	**1.48× 10** ^**4**^	**3.39× 10** ^**5**^	**2.54× 10** ^**6**^	**2.43× 10** ^**6**^	**2.98× 10** ^**7**^	**1.67× 10** ^**5**^	**3.51× 10** ^**5**^	**2.82× 10** ^**5**^	**2.32× 10** ^**6**^	**7.44× 10** ^**6**^
	Best	1.29× 10^4^	6.40× 10^4^	1.45× 10^6^	5.52× 10^5^	2.46× 10^6^	8.15× 10^4^	3.62× 10^4^	**2.91× 10** ^**3**^	**9.84× 10** ^**4**^	**7.02× 10** ^**6**^
	Rank	2	4	8	7	9	5	3	**1**	6	10
F6	Mean	**1.98× 10** ^**3**^	**2.01× 10** ^**3**^	**3.40× 10** ^**3**^	**3.01× 10** ^**3**^	**3.65× 10** ^**3**^	**2.59× 10** ^**3**^	**2.51× 10** ^**3**^	**2.18× 10** ^**3**^	**2.27× 10** ^**3**^	**4.51× 10** ^**3**^
	Std	**1.45× 10** ^**2**^	**1.69× 10** ^**2**^	**2.13× 10** ^**2**^	**3.39× 10** ^**2**^	**7.18× 10** ^**2**^	**3.31× 10** ^**2**^	**2.74× 10** ^**2**^	**2.53× 10** ^**2**^	**2.95× 10** ^**2**^	**3.19× 10** ^**2**^
	Best	1.73× 10^3^	1.78× 10^3^	3.00× 10^3^	2.52× 10^3^	2.62× 10^3^	1.94× 10^3^	2.02× 10^3^	**1.68× 10** ^**3**^	**1.80× 10** ^**3**^	**3.78× 10** ^**3**^
	Rank	2	3	9	7	8	5	6	**1**	4	10
F7	Mean	1.81× 10^4^	1.91× 10^5^	1.25× 10^6^	1.28× 10^6^	1.19× 10^7^	1.11× 10^5^	**7.40× 10** ^**4**^	**3.75× 10** ^**3**^	**4.05× 10** ^**5**^	**6.88× 10** ^**6**^
	Std	8.22× 10^3^	2.01× 10^5^	8.08× 10^5^	1.51× 10^6^	1.01× 10^7^	5.71× 10^4^	3.93× 10^4^	**9.98× 10** ^**2**^	**4.17× 10** ^**5**^	**2.90× 10** ^**6**^
	Best	6.98× 10^3^	1.82× 10^4^	2.04× 10^5^	5.65× 10^4^	6.31× 10^5^	2.47× 10^4^	1.68× 10^4^	**2.57× 10** ^**3**^	**6.66× 10** ^**3**^	**2.14× 10** ^**6**^
	Rank	3	5	8	7	9	6	4	**1**	2	10
F8	Mean	**2.30× 10** ^**3**^	**2.41× 10** ^**3**^	**7.66× 10** ^**3**^	**6.03× 10** ^**3**^	**8.38× 10** ^**3**^	**3.94× 10** ^**3**^	**4.93× 10** ^**3**^	**2.68× 10** ^**3**^	**5.17× 10** ^**3**^	**8.43× 10** ^**3**^
	Std	**3.68**	**6.01× 10** ^**2**^	**2.86× 10** ^**3**^	**2.30× 10** ^**3**^	**9.32× 10** ^**2**^	**2.52× 10** ^**3**^	**2.36× 10** ^**3**^	**2.36× 10** ^**2**^	**1.72× 10** ^**3**^	**6.71× 10** ^**2**^
	Best	2.30× 10^3^	2.30× 10^3^	3.26× 10^3^	2.31× 10^3^	6.05× 10^3^	2.41× 10^3^	2.32× 10^3^	2.43× 10^3^	**2.30× 10** ^**3**^	**6.67× 10** ^**3**^
	Rank	2	3	8	4	9	6	5	7	**1**	10
F9	Mean	**2.94× 10** ^**3**^	**2.94× 10** ^**3**^	**3.16× 10** ^**3**^	**3.18× 10** ^**3**^	**3.48× 10** ^**3**^	**3.09× 10** ^**3**^	**3.32× 10** ^**3**^	**3.00× 10** ^**3**^	**3.02× 10** ^**3**^	**3.50× 10** ^**3**^
	Std	4.83$\times 10^{1}$	3.45$\times 10^{1}$	**3.08$\times 10^{1}$**	**8.55$\times 10^{1}$**	**1.16× 10** ^**2**^	**7.25$\times 10^{1}$**	**1.23× 10** ^**2**^	**7.54$\times 10^{1}$**	**4.59$\times 10^{1}$**	**6.18$\times 10^{1}$**
	Best	**2.87× 10** ^**3**^	**2.90× 10** ^**3**^	**3.10× 10** ^**3**^	**3.00× 10** ^**3**^	**3.27× 10** ^**3**^	**2.96× 10** ^**3**^	**3.09× 10** ^**3**^	**2.88× 10** ^**3**^	**2.93× 10** ^**3**^	**3.33× 10** ^**3**^
	Rank	**1**	3	8	6	9	5	7	2	4	10
F10	Mean	**2.91× 10** ^**3**^	**2.91× 10** ^**3**^	**3.20× 10** ^**3**^	**2.95× 10** ^**3**^	**4.13× 10** ^**3**^	**2.98× 10** ^**3**^	**2.89× 10** ^**3**^	**3.05× 10** ^**3**^	**2.92× 10** ^**3**^	**6.58× 10** ^**3**^
	Std	2.05$\times 10^{1}$	2.37$\times 10^{1}$	8.21$\times 10^{1}$	2.83$\times 10^{1}$	3.76× 10^2^	1.29$\times 10^{1}$	**4.10**	**4.95$\times 10^{1}$**	**4.04$\times 10^{1}$**	**8.48× 10** ^**2**^
	Best	**2.88× 10** ^**3**^	**2.88× 10** ^**3**^	**3.11× 10** ^**3**^	**2.90× 10** ^**3**^	**3.52× 10** ^**3**^	**2.96× 10** ^**3**^	**2.88× 10** ^**3**^	**2.96× 10** ^**3**^	**2.89× 10** ^**3**^	**4.52× 10** ^**3**^
	Rank	**1**	2	8	5	9	6	3	7	4	10
Average	Mean	**7.69× 10** ^**3**^	**7.89× 10** ^**4**^	**1.23× 10** ^**12**^	**7.64× 10** ^**5**^	**3.72× 10** ^**12**^	**9.66× 10** ^**6**^	**1.51× 10** ^**6**^	**2.77× 10** ^**8**^	**4.34× 10** ^**5**^	**5.87× 10** ^**12**^
	Std	**3.70× 10** ^**3**^	**1.08× 10** ^**5**^	**1.73× 10** ^**11**^	**5.68× 10** ^**5**^	**8.35× 10** ^**11**^	**5.58× 10** ^**6**^	**2.10× 10** ^**5**^	**1.24× 10** ^**8**^	**1.19× 10** ^**6**^	**4.74× 10** ^**11**^
	Best	**3.65× 10** ^**3**^	**9.96× 10** ^**3**^	**9.09× 10** ^**11**^	**1.24× 10** ^**5**^	**1.79× 10** ^**12**^	**1.86× 10** ^**6**^	**1.06× 10** ^**6**^	**9.45× 10** ^**7**^	**1.21× 10** ^**4**^	**4.91× 10** ^**12**^
	Rank	**1**	2	8	4	9	6	5	7	3	10

Overall, the average ranking across all 30-dimensional tests places ICOA at the forefront among the evaluated algorithms, further confirming its superiority in high-dimensional global optimization problems. The comprehensive experimental results reveal that ICOA effectively balances global exploration and local exploitation in high-dimensional scenarios, achieves rapid convergence with stable outcomes, and exhibits strong adaptability across diverse objective function types.

**Fig 8:**
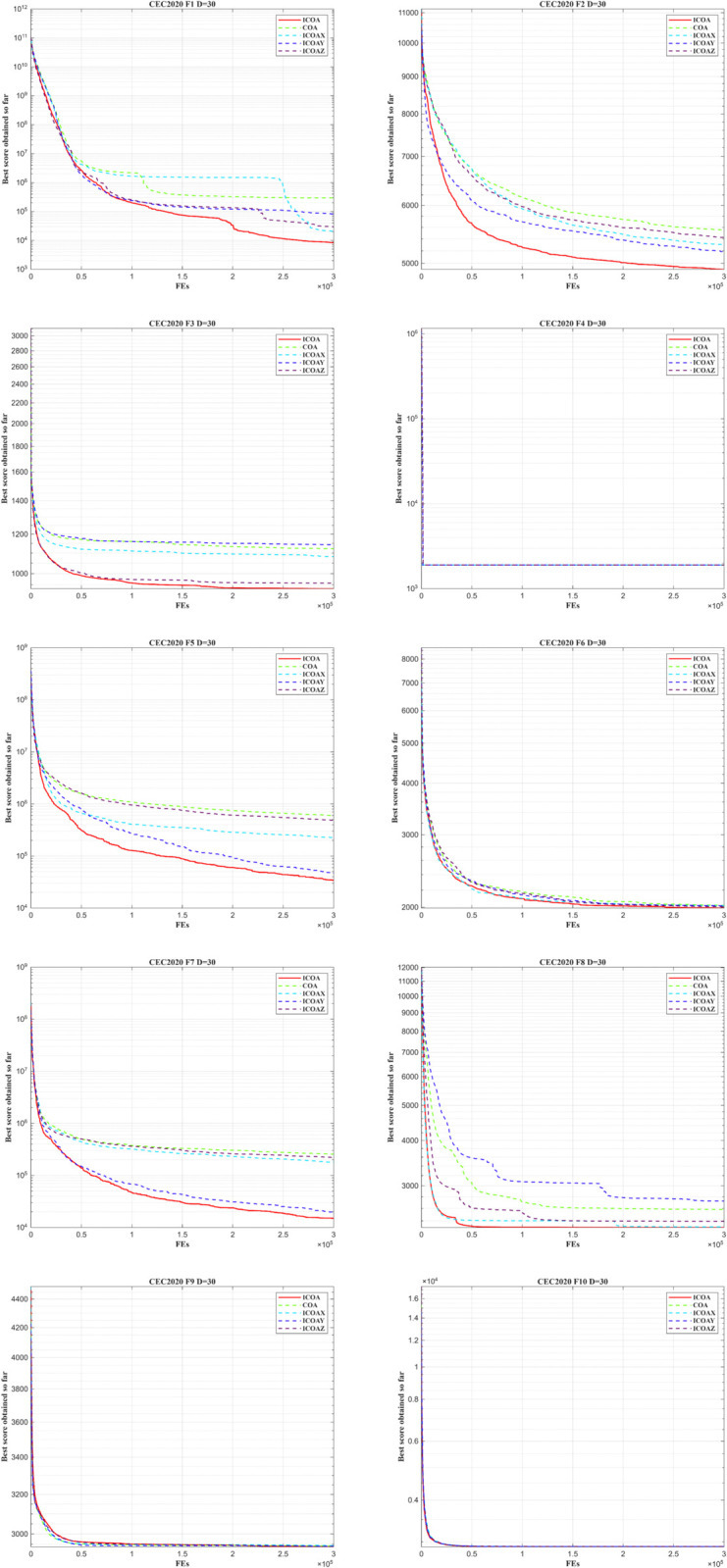
Convergence curve of ICOA in IEEE CEC2020 (DIM=30).

ICOA exhibits excellent convergence precision and fast convergence in all functions in Fig 8. Furthermore, in the comparison of convergence between 20 and 30 dimensions, it is clear that ICOA consistently produces excellent optimization results. It can be inferred that the strategy developed in this work continues to perform efficiently with increasing dimensions.

**Fig 9:**
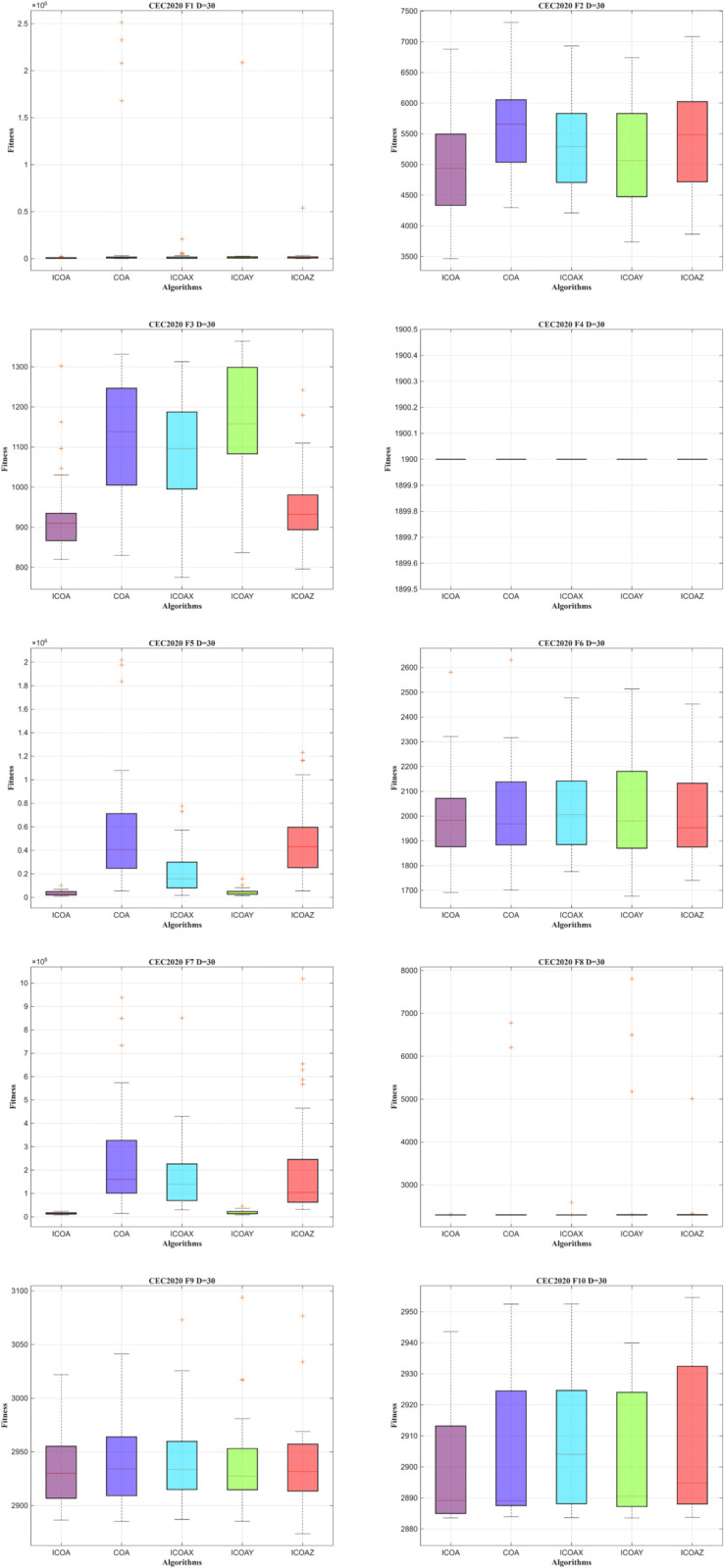
Box plot of ICOA in IEEE CEC2020 (DIM=30).

The box plots in Fig 9 illustrate that ICOA generates solutions with a more concentrated distribution and superior quality. This shows its strong performance in balancing global search and local search spaces, consistently yielding positive results.

### 4.6 CEC2020(DIM=50) benchmark test function experiment

As shown in Table 9, when the dimensionality increases from 20 to 50, the overall optimization difficulty rises significantly, with the mean fitness values (Mean) and best fitness values (Best) of all algorithms generally increasing. This indicates a substantial increase in both search complexity and the number of local optima in high-dimensional spaces. Under these conditions, the ICOA algorithm still demonstrates strong stability and global search capability. Compared to the 20-dimensional results, although ICOA experiences a slight increase in fitness values in the 50-dimensional tests, its performance degradation is smaller than that of most comparative algorithms. Particularly in complex multimodal functions, it maintains lower average errors and higher solution convergence precision, reflecting excellent high-dimensional robustness. From the perspective of the standard deviation (Std) metric, ICOA exhibits relatively stable fluctuation amplitudes in high-dimensional scenarios, indicating that the algorithm can still maintain consistent search results across multiple independent runs without significant instability. Overall, ICOA demonstrates good scalability in cross-dimensional tests from 20 to 50. When facing the increased complexity introduced by expanding the search space, it maintains a good balance between exploration and exploitation, exhibiting superior global optimization capability and convergence stability compared to other algorithms.

**Table 9:** Experimental outcomes for each algorithm in IEEE2020 D=50 (optimal data are highlighted in bold).

Functions	Statistics	ICOA	COA	SCA	WOA	HHO	WDO	PSO	APO	DBO	BKA
F1	Mean	**1.68× 10** ^**6**^	**3.59× 10** ^**6**^	**3.98× 10** ^**10**^	**7.62× 10** ^**6**^	**9.15× 10** ^**10**^	**4.45× 10** ^**8**^	**1.33× 10** ^**8**^	**5.72× 10** ^**9**^	**2.51× 10** ^**9**^	**1.42× 10** ^**11**^
	Std	**3.15× 10** ^**6**^	**1.10× 10** ^**7**^	**5.90× 10** ^**9**^	**5.80× 10** ^**6**^	**1.29× 10** ^**10**^	**1.11× 10** ^**8**^	**3.46× 10** ^**8**^	**2.16× 10** ^**9**^	**1.38× 10** ^**10**^	**1.10× 10** ^**10**^
	Best	2.12× 10^4^	4.77× 10^4^	2.21× 10^10^	1.86× 10^6^	6.59× 10^10^	2.38× 10^8^	3.13× 10^7^	2.37× 10^9^	**5.25× 10** ^**2**^	**1.20× 10** ^**11**^
	Rank	2	3	8	4	9	6	5	7	**1**	10
F2	Mean	**8.19× 10** ^**3**^	**8.37× 10** ^**3**^	**1.46× 10** ^**4**^	**1.01× 10** ^**4**^	**1.35× 10** ^**4**^	**9.55× 10** ^**3**^	**9.11× 10** ^**3**^	**8.86× 10** ^**3**^	**8.63× 10** ^**3**^	**1.50× 10** ^**4**^
	Std	6.45× 10^2^	7.44× 10^2^	**4.15× 10** ^**2**^	**1.28× 10** ^**3**^	**7.38× 10** ^**2**^	**1.02× 10** ^**3**^	**9.90× 10** ^**2**^	**5.62× 10** ^**2**^	**9.26× 10** ^**2**^	**4.22× 10** ^**2**^
	Best	6.87× 10^3^	**6.52× 10** ^**3**^	**1.39× 10** ^**4**^	**8.04× 10** ^**3**^	**1.17× 10** ^**4**^	**7.76× 10** ^**3**^	**7.04× 10** ^**3**^	**7.64× 10** ^**3**^	**6.83× 10** ^**3**^	**1.39× 10** ^**4**^
	Rank	3	**1**	10	7	8	6	4	5	2	9
F3	Mean	1.44× 10^3^	1.69× 10^3^	1.60× 10^3^	1.69× 10^3^	1.90× 10^3^	**1.35× 10** ^**3**^	**1.47× 10** ^**3**^	**1.55× 10** ^**3**^	**1.36× 10** ^**3**^	**2.96× 10** ^**3**^
	Std	2.74× 10^2^	1.21× 10^2^	7.34$\times 10^{1}$	1.14× 10^2^	7.07$\times 10^{1}$	8.78$\times 10^{1}$	**6.81$\times 10^{1}$**	**1.84× 10** ^**2**^	**2.71× 10** ^**2**^	**2.39× 10** ^**2**^
	Best	**9.38× 10** ^**2**^	**1.37× 10** ^**3**^	**1.44× 10** ^**3**^	**1.44× 10** ^**3**^	**1.76× 10** ^**3**^	**1.13× 10** ^**3**^	**1.35× 10** ^**3**^	**1.29× 10** ^**3**^	**1.02× 10** ^**3**^	**2.46× 10** ^**3**^
	Rank	**1**	6	7	8	9	3	5	4	2	10
F4	Mean	**1.90× 10** ^**3**^	**1.90× 10** ^**3**^	**1.90× 10** ^**3**^	**1.90× 10** ^**3**^	**1.90× 10** ^**3**^	**1.90× 10** ^**3**^	**1.93× 10** ^**3**^	**1.90× 10** ^**3**^	**2.07× 10** ^**3**^	**2.61× 10** ^**3**^
	Std	**0.00**	**0.00**	**5.44**	**0.00**	**0.00**	**5.14**	**2.21**	**0.00**	**3.89× 10** ^**2**^	**6.31× 10** ^**2**^
	Best	**1.90× 10** ^**3**^	**1.90× 10** ^**3**^	**1.90× 10** ^**3**^	**1.90× 10** ^**3**^	**1.90× 10** ^**3**^	**1.90× 10** ^**3**^	**1.93× 10** ^**3**^	**1.90× 10** ^**3**^	**1.91× 10** ^**3**^	**1.98× 10** ^**3**^
	Rank	**1**	**1**	**1**	**1**	**1**	**1**	9	**1**	8	10
F5	Mean	**1.76× 10** ^**5**^	**8.56× 10** ^**5**^	**3.51× 10** ^**7**^	**1.67× 10** ^**7**^	**3.34× 10** ^**8**^	**9.12× 10** ^**5**^	**6.07× 10** ^**5**^	**6.02× 10** ^**5**^	**6.20× 10** ^**6**^	**1.78× 10** ^**8**^
	Std	**7.00× 10** ^**4**^	**5.98× 10** ^**5**^	**1.19× 10** ^**7**^	**9.00× 10** ^**6**^	**1.95× 10** ^**8**^	**2.92× 10** ^**5**^	**3.59× 10** ^**5**^	**5.27× 10** ^**5**^	**6.39× 10** ^**6**^	**5.64× 10** ^**7**^
	Best	**6.04× 10** ^**4**^	**1.46× 10** ^**5**^	**1.53× 10** ^**7**^	**3.04× 10** ^**6**^	**3.43× 10** ^**7**^	**2.35× 10** ^**5**^	**1.72× 10** ^**5**^	**1.03× 10** ^**5**^	**1.55× 10** ^**5**^	**6.78× 10** ^**7**^
	Rank	**1**	3	8	7	9	6	5	2	4	10
F6	Mean	**2.76× 10** ^**3**^	**2.79× 10** ^**3**^	**5.74× 10** ^**3**^	**5.23× 10** ^**3**^	**6.69× 10** ^**3**^	**4.05× 10** ^**3**^	**3.75× 10** ^**3**^	**3.14× 10** ^**3**^	**3.45× 10** ^**3**^	**8.02× 10** ^**3**^
	Std	3.62× 10^2^	3.81× 10^2^	4.99× 10^2^	8.27× 10^2^	8.96× 10^2^	5.42× 10^2^	4.71× 10^2^	**3.06× 10** ^**2**^	**4.58× 10** ^**2**^	**4.79× 10** ^**2**^
	Best	**2.17× 10** ^**3**^	**2.28× 10** ^**3**^	**4.48× 10** ^**3**^	**3.56× 10** ^**3**^	**5.03× 10** ^**3**^	**2.83× 10** ^**3**^	**2.90× 10** ^**3**^	**2.35× 10** ^**3**^	**2.37× 10** ^**3**^	**7.06× 10** ^**3**^
	Rank	**1**	2	8	7	9	5	6	3	4	10
F7	Mean	**1.12× 10** ^**5**^	**4.72× 10** ^**5**^	**8.86× 10** ^**6**^	**6.69× 10** ^**6**^	**5.07× 10** ^**7**^	**4.22× 10** ^**5**^	**2.44× 10** ^**5**^	**1.41× 10** ^**5**^	**2.64× 10** ^**6**^	**4.21× 10** ^**7**^
	Std	**4.65× 10** ^**4**^	**2.69× 10** ^**5**^	**3.88× 10** ^**6**^	**4.72× 10** ^**6**^	**3.25× 10** ^**7**^	**1.24× 10** ^**5**^	**1.15× 10** ^**5**^	**1.13× 10** ^**5**^	**3.60× 10** ^**6**^	**1.03× 10** ^**7**^
	Best	4.33× 10^4^	8.65× 10^4^	2.60× 10^6^	1.76× 10^6^	9.89× 10^6^	1.67× 10^5^	7.00× 10^4^	**1.34× 10** ^**4**^	**8.14× 10** ^**4**^	**2.09× 10** ^**7**^
	Rank	2	5	8	7	9	6	3	**1**	4	10
F8	Mean	**9.81× 10** ^**3**^	**1.09× 10** ^**4**^	**1.56× 10** ^**4**^	**1.18× 10** ^**4**^	**1.48× 10** ^**4**^	**1.11× 10** ^**4**^	**1.04× 10** ^**4**^	**9.87× 10** ^**3**^	**1.02× 10** ^**4**^	**1.63× 10** ^**4**^
	Std	3.15× 10^3^	2.58× 10^3^	1.48× 10^3^	1.32× 10^3^	7.28× 10^2^	1.89× 10^3^	9.95× 10^2^	2.07× 10^3^	9.64× 10^2^	**3.98× 10** ^**2**^
	Best	**2.30× 10** ^**3**^	**2.30× 10** ^**3**^	**7.95× 10** ^**3**^	**9.41× 10** ^**3**^	**1.30× 10** ^**4**^	**2.37× 10** ^**3**^	**7.83× 10** ^**3**^	**3.27× 10** ^**3**^	**8.52× 10** ^**3**^	**1.53× 10** ^**4**^
	Rank	**1**	2	6	8	9	3	5	4	7	10
F9	Mean	**3.22× 10** ^**3**^	**3.23× 10** ^**3**^	**3.66× 10** ^**3**^	**3.65× 10** ^**3**^	**4.38× 10** ^**3**^	**3.53× 10** ^**3**^	**3.77× 10** ^**3**^	**3.42× 10** ^**3**^	**3.38× 10** ^**3**^	**4.39× 10** ^**3**^
	Std	9.46$\times 10^{1}$	1.10× 10^2^	**5.09$\times 10^{1}$**	**1.53× 10** ^**2**^	**2.42× 10** ^**2**^	**1.06× 10** ^**2**^	**1.61× 10** ^**2**^	**1.18× 10** ^**2**^	**1.15× 10** ^**2**^	**9.79$\times 10^{1}$**
	Best	**3.06× 10** ^**3**^	**3.08× 10** ^**3**^	**3.57× 10** ^**3**^	**3.38× 10** ^**3**^	**3.93× 10** ^**3**^	**3.27× 10** ^**3**^	**3.43× 10** ^**3**^	**3.25× 10** ^**3**^	**3.18× 10** ^**3**^	**4.11× 10** ^**3**^
	Rank	**1**	2	8	6	9	5	7	4	3	10
F10	Mean	3.08× 10^3^	3.08× 10^3^	6.00× 10^3^	3.14× 10^3^	1.25× 10^4^	3.14× 10^3^	**3.06× 10** ^**3**^	**3.80× 10** ^**3**^	**3.33× 10** ^**3**^	**2.33× 10** ^**4**^
	Std	2.98$\times 10^{1}$	4.46$\times 10^{1}$	4.15× 10^2^	4.35$\times 10^{1}$	1.55× 10^3^	**1.65$\times 10^{1}$**	**3.42$\times 10^{1}$**	**3.29× 10** ^**2**^	**1.23× 10** ^**3**^	**2.94× 10** ^**3**^
	Best	3.02× 10^3^	2.97× 10^3^	5.26× 10^3^	3.05× 10^3^	9.10× 10^3^	3.11× 10^3^	**2.97× 10** ^**3**^	**3.34× 10** ^**3**^	**3.02× 10** ^**3**^	**1.52× 10** ^**4**^
	Rank	3	2	8	5	9	6	**1**	7	4	10
Average	Mean	**2.00× 10** ^**5**^	**4.95× 10** ^**5**^	**3.98× 10** ^**9**^	**3.10× 10** ^**6**^	**9.18× 10** ^**9**^	**4.47× 10** ^**7**^	**1.34× 10** ^**7**^	**5.72× 10** ^**8**^	**2.52× 10** ^**8**^	**1.42× 10** ^**10**^
	Std	**3.27× 10** ^**5**^	**1.19× 10** ^**6**^	**5.92× 10** ^**8**^	**2.05× 10** ^**6**^	**1.31× 10** ^**9**^	**1.11× 10** ^**7**^	**3.46× 10** ^**7**^	**2.16× 10** ^**8**^	**1.38× 10** ^**9**^	**1.11× 10** ^**9**^
	Best	**1.45× 10** ^**4**^	**3.00× 10** ^**4**^	**2.21× 10** ^**9**^	**6.69× 10** ^**5**^	**6.59× 10** ^**9**^	**2.39× 10** ^**7**^	**3.15× 10** ^**6**^	**2.37× 10** ^**8**^	**2.64× 10** ^**4**^	**1.20× 10** ^**10**^
	Rank	**1**	3	8	4	9	6	5	7	2	10

**Fig 10:**
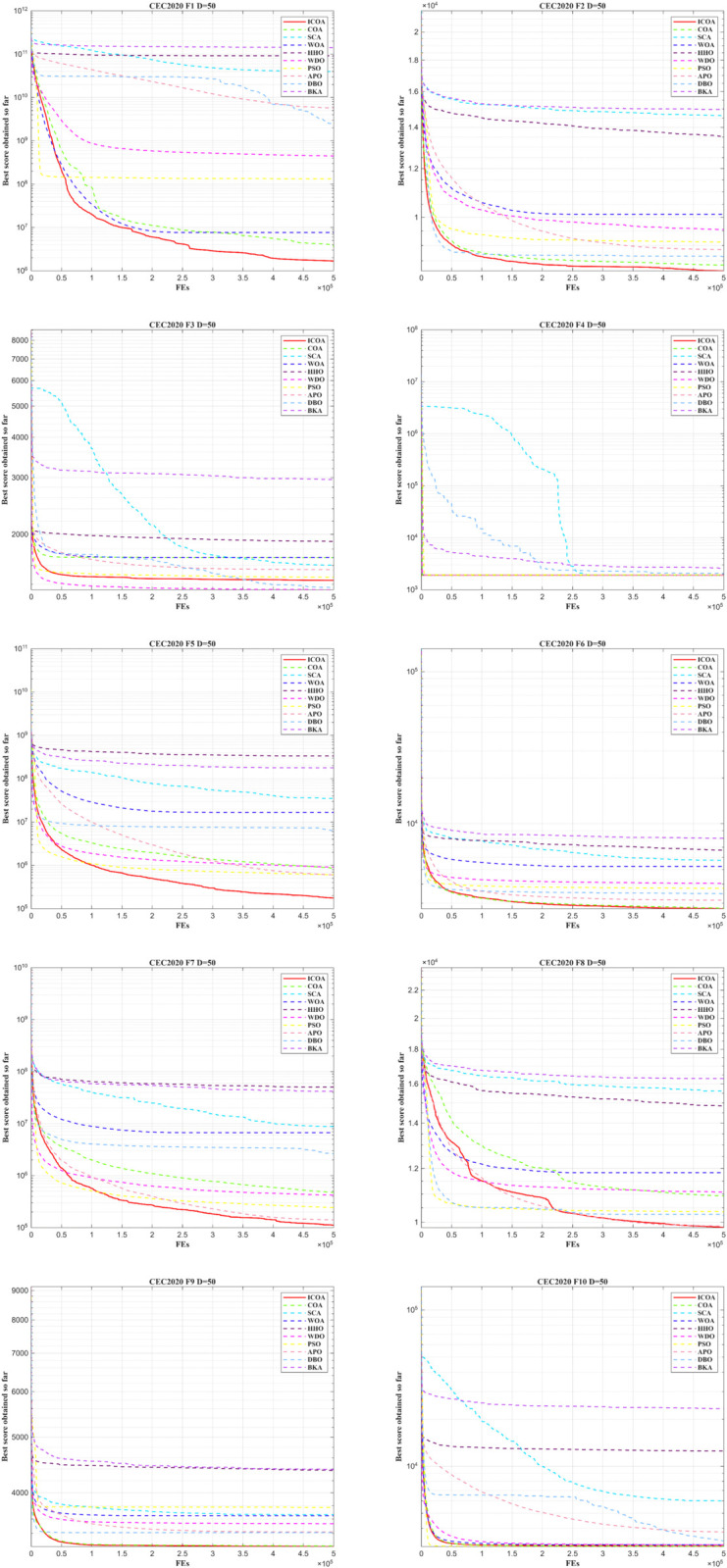
Convergence curve of ICOA in IEEE CEC2020 (DIM=50).

**Fig 11:**
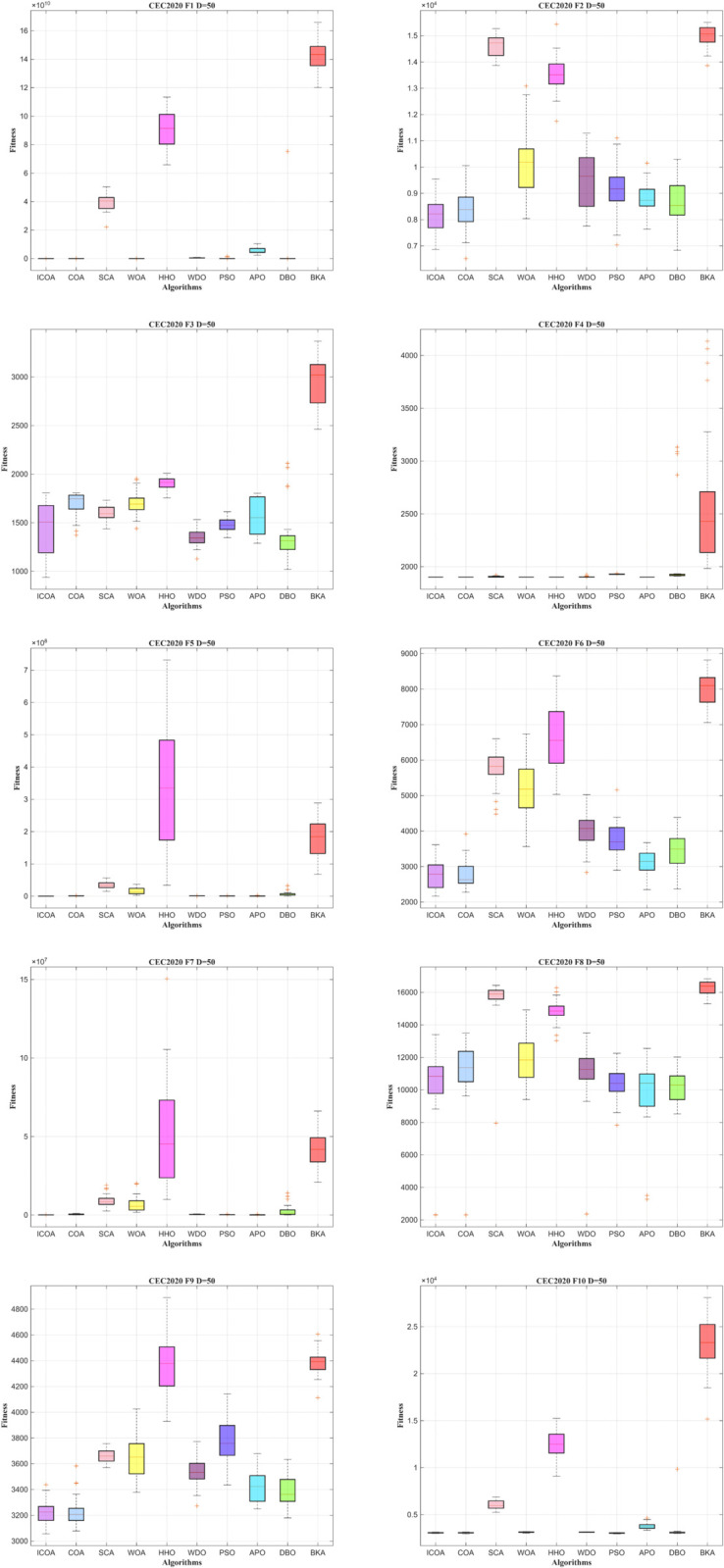
Comparison of box diagrams of the 10 algorithms using CEC2020 (Dim=50).

In Figs 10 and 11, ICOA achieves faster convergence with higher accuracy in the remaining functions. This suggests that ICOA demonstrates faster convergence performance. Clearly, the solutions obtained by ICOA exhibit a more concentrated distribution with fewer poor values, highlighting the improved robustness of ICOA.

### 4.7 Nonparametric statistical analysis

#### 4.7.1 Effectiveness analysis of the ICOA

To verify the effectiveness of the core strategy in the improved crayfish optimization algorithm, ablation experiments were conducted on the CEC2020 test set with a 30-dimensional scenario. The experiment designed five comparative schemes: 1) the complete ICOA algorithm (integrating Sobol sequence initialization, Levy flight, and Euclidean distance-fitness balanced competition strategy); 2) the original COA algorithm; 3) ICOAX (removing Sobol sequence initialization and adopting random initialization); 4) ICOAY (removing Levy flight and retaining the basic search mechanism); 5) ICOAZ (removing the Euclidean distance-fitness balanced competition strategy and adopting traditional competition selection rul optimization accuracy (mean, standard deviation, and optimal value) of the five algorithms on the CEC2020 test set functions was used as the core evaluation metric. The experimental results showed that the complete ICOA algorithm exhibited comprehensive advantages across 10 test functions.

Table 11 presents the ablation study based on the CEC2020 test set. The parameter settings of all algorithms follow those of the COA algorithm, with a population size of 100 and a maximum of 10,000 iterations. As shown in Table 10, for the unimodal function F1, each strategy yields a noticeable improvement in optimization performance compared with the original COA algorithm, while the combined strategies achieve the best overall enhancement. For the basic functions (F2–F4), ICOA exhibits a substantial improvement on F2 and is able to reach the optimal value; a moderate improvement is observed on F3, and similar optimization results are obtained on F4. For the hybrid functions (F5–F7) and composition functions (F8–F10), all strategies contribute to better optimization performance and improved stability. Overall, the ablation study demonstrates that each strategy enhances the search capability of the original COA, and the integrated ICOA achieves superior performance, confirming the effectiveness and feasibility of the proposed improvements.

**Table 10:** The overall runtime comparison of ICOA across different dimensions of the CEC2020 benchmark functions.

Function	ICOA			COA			SCA			WOA			HHO		
Dim	D=20	D=30	D=50	D=20	D=30	D=50	D=20	D=30	D=50	D=20	D=30	D=50	D=20	D=30	D=50
F1	4.04	7.80	19.05	0.98	2.20	6.75	1.35	3.03	9.02	0.76	1.62	4.50	1.88	3.98	13.21
F2	4.78	9.27	23.09	1.63	3.62	10.50	1.78	3.98	11.45	1.15	2.39	6.85	3.00	6.34	20.29
F3	4.64	8.75	22.29	1.50	3.19	9.91	1.62	3.64	10.53	1.04	2.14	6.09	2.64	5.53	17.57
F4	4.64	8.73	20.06	1.36	2.66	6.41	1.46	3.07	7.89	0.87	1.65	3.62	2.30	4.39	11.14
F5	4.58	8.93	22.21	1.51	3.40	10.12	1.65	3.85	11.05	1.06	2.30	6.51	2.70	5.97	19.01
F6	4.39	8.49	20.87	1.32	2.92	8.68	1.58	3.55	10.23	0.96	2.04	5.76	2.45	5.26	16.77
F7	4.52	8.57	21.28	1.38	2.93	9.14	1.61	3.55	10.46	1.00	2.07	6.02	2.56	5.36	17.33
F8	5.97	12.96	37.26	2.89	7.24	24.36	2.51	6.26	19.61	1.91	4.74	15.06	4.88	12.07	42.80
F9	6.68	14.66	44.28	3.55	9.02	31.30	2.88	7.24	23.98	2.27	5.70	19.08	5.80	14.51	53.11
F10	5.94	13.37	41.30	2.88	7.70	28.78	2.45	6.49	22.42	1.89	4.99	17.73	4.76	12.58	49.43
AVGTime	5.02	10.15	27.17	1.90	4.49	14.59	1.89	4.47	13.66	1.29	2.96	9.12	3.30	7.60	26.07
Function	**WDO**			**PSO**			**APO**			**DBO**			**BKA**		
Dim	D=20	D=30	D=50	D=20	D=30	D=50	D=20	D=30	D=50	D=20	D=30	D=50	D=20	D=30	D=50
F1	1.31	2.54	6.72	0.94	1.92	5.30	1.59	3.38	10.05	1.41	2.60	6.47	0.64	1.45	4.95
F2	1.76	3.39	8.80	1.35	2.79	7.61	2.36	5.00	14.32	1.91	3.58	8.83	1.04	2.29	7.66
F3	1.66	3.11	8.16	1.22	2.50	6.78	2.15	4.50	12.78	1.72	3.14	7.97	0.91	2.02	6.71
F4	1.43	2.54	5.60	1.05	1.94	4.31	1.80	3.45	7.78	1.51	2.64	5.41	0.76	1.47	3.79
F5	1.61	3.31	8.72	1.24	2.59	7.35	2.19	4.83	13.98	1.84	3.58	8.89	0.94	2.12	7.22
F6	1.50	3.03	7.83	1.13	2.34	6.38	1.99	4.24	12.13	1.64	3.22	7.69	0.84	1.84	6.21
F7	1.61	3.04	8.10	1.18	2.41	6.68	2.06	4.34	12.84	1.88	3.36	8.15	0.87	1.90	6.52
F8	2.46	5.59	17.37	2.09	5.00	15.78	3.88	9.52	31.11	2.65	5.87	17.16	1.81	4.52	17.05
F9	2.85	6.72	21.50	2.46	6.06	20.09	4.62	11.48	39.24	2.96	6.70	21.14	2.16	5.43	21.40
F10	2.44	5.96	19.88	2.08	5.38	18.83	3.82	10.14	36.44	2.55	6.18	20.15	1.75	4.70	19.80
AVGTime	1.86	3.92	11.27	1.47	3.29	9.91	2.64	6.09	19.07	2.01	4.09	11.19	1.17	2.77	10.13

**Table 11:** Ablation study results on CEC2020.

Functions	Statistics	ICOA	COA	ICOAX	ICOAY	ICOAZ
F1	Best	**3.84× 10** ^**2**^	**6.10× 10** ^**2**^	**2.01× 10** ^**3**^	**2.75× 10** ^**3**^	**1.44× 10** ^**3**^
	Mean	**8.33× 10** ^**3**^	**2.96× 10** ^**5**^	**2.07× 10** ^**4**^	**8.20× 10** ^**4**^	**2.97× 10** ^**4**^
	Std	**7.09× 10** ^**3**^	**7.49× 10** ^**5**^	**3.89× 10** ^**4**^	**3.79× 10** ^**5**^	**9.65× 10** ^**4**^
	Rank	**1**	2	4	5	3
F2	Best	**3.47× 10** ^**3**^	**4.30× 10** ^**3**^	**4.21× 10** ^**3**^	**3.74× 10** ^**3**^	**3.86× 10** ^**3**^
	Mean	**4.91× 10** ^**3**^	**5.55× 10** ^**3**^	**5.30× 10** ^**3**^	**5.18× 10** ^**3**^	**5.43× 10** ^**3**^
	Std	9.14× 10^2^	7.44× 10^2^	**7.36× 10** ^**2**^	**8.06× 10** ^**2**^	**8.51× 10** ^**2**^
	Rank	**1**	5	4	2	3
F3	Best	8.20× 10^2^	8.29× 10^2^	**7.75× 10** ^**2**^	**8.37× 10** ^**2**^	**7.96× 10** ^**2**^
	Mean	**9.33× 10** ^**2**^	**1.12× 10** ^**3**^	**1.08× 10** ^**3**^	**1.14× 10** ^**3**^	**9.57× 10** ^**2**^
	Std	1.04× 10^2^	1.36× 10^2^	1.37× 10^2^	1.66× 10^2^	**1.00× 10** ^**2**^
	Rank	3	4	**1**	5	2
F4	Best	**1.90× 10** ^**3**^	**1.90× 10** ^**3**^	**1.90× 10** ^**3**^	**1.90× 10** ^**3**^	**1.90× 10** ^**3**^
	Mean	**1.90× 10** ^**3**^	**1.90× 10** ^**3**^	**1.90× 10** ^**3**^	**1.90× 10** ^**3**^	**1.90× 10** ^**3**^
	Std	**0.00**	**0.00**	**0.00**	**0.00**	**0.00**
	Rank	**1**	**1**	**1**	**1**	**1**
F5	Best	**1.00× 10** ^**4**^	**5.49× 10** ^**4**^	**1.73× 10** ^**4**^	**1.37× 10** ^**4**^	**5.52× 10** ^**4**^
	Mean	**3.42× 10** ^**4**^	**5.96× 10** ^**5**^	**2.26× 10** ^**5**^	**4.67× 10** ^**4**^	**4.87× 10** ^**5**^
	Std	**2.03× 10** ^**4**^	**5.28× 10** ^**5**^	**2.00× 10** ^**5**^	**2.92× 10** ^**4**^	**3.19× 10** ^**5**^
	Rank	**1**	4	3	2	5
F6	Best	1.69× 10^3^	1.70× 10^3^	1.78× 10^3^	**1.68× 10** ^**3**^	**1.74× 10** ^**3**^
	Mean	**1.99× 10** ^**3**^	**2.02× 10** ^**3**^	**2.02× 10** ^**3**^	**2.02× 10** ^**3**^	**2.01× 10** ^**3**^
	Std	1.85× 10^2^	2.00× 10^2^	**1.61× 10** ^**2**^	**2.22× 10** ^**2**^	**1.81× 10** ^**2**^
	Rank	2	3	5	**1**	4
F7	Best	**8.25× 10** ^**3**^	**1.44× 10** ^**4**^	**3.05× 10** ^**4**^	**8.84× 10** ^**3**^	**3.16× 10** ^**4**^
	Mean	**1.50× 10** ^**4**^	**2.57× 10** ^**5**^	**1.78× 10** ^**5**^	**1.97× 10** ^**4**^	**2.21× 10** ^**5**^
	Std	**4.21× 10** ^**3**^	**2.41× 10** ^**5**^	**1.61× 10** ^**5**^	**8.43× 10** ^**3**^	**2.44× 10** ^**5**^
	Rank	**1**	3	4	2	5
F8	Best	2.30× 10^3^	**2.30× 10** ^**3**^	**2.30× 10** ^**3**^	**2.30× 10** ^**3**^	**2.30× 10** ^**3**^
	Mean	**2.30× 10** ^**3**^	**2.58× 10** ^**3**^	**2.31× 10** ^**3**^	**2.72× 10** ^**3**^	**2.39× 10** ^**3**^
	Std	**2.68**	**1.07× 10** ^**3**^	**5.29$\times 10^{1}$**	**1.32× 10** ^**3**^	**4.95× 10** ^**2**^
	Rank	5	**1**	3	2	4
F9	Best	2.89× 10^3^	2.89× 10^3^	2.89× 10^3^	2.89× 10^3^	**2.87× 10** ^**3**^
	Mean	**2.94× 10** ^**3**^	**2.94× 10** ^**3**^	**2.94× 10** ^**3**^	**2.94× 10** ^**3**^	**2.94× 10** ^**3**^
	Std	**3.85$\times 10^{1}$**	**4.21$\times 10^{1}$**	**4.60$\times 10^{1}$**	**4.32$\times 10^{1}$**	**4.04$\times 10^{1}$**
	Rank	4	2	5	3	**1**
F10	Best	**2.88× 10** ^**3**^	**2.88× 10** ^**3**^	**2.88× 10** ^**3**^	**2.88× 10** ^**3**^	**2.88× 10** ^**3**^
	Mean	**2.90× 10** ^**3**^	**2.90× 10** ^**3**^	**2.91× 10** ^**3**^	**2.90× 10** ^**3**^	**2.91× 10** ^**3**^
	Std	**1.97$\times 10^{1}$**	**2.19$\times 10^{1}$**	**2.13$\times 10^{1}$**	**2.09$\times 10^{1}$**	**2.34$\times 10^{1}$**
	Rank	2	5	3	**1**	4
Average	Best	**3.46× 10** ^**3**^	**8.67× 10** ^**3**^	**6.65× 10** ^**3**^	**4.15× 10** ^**3**^	**1.05× 10** ^**4**^
	Mean	**7.55× 10** ^**3**^	**1.17× 10** ^**5**^	**4.43× 10** ^**4**^	**1.67× 10** ^**4**^	**7.56× 10** ^**4**^
	Std	**3.28× 10** ^**3**^	**1.52× 10** ^**5**^	**4.02× 10** ^**4**^	**4.19× 10** ^**4**^	**6.61× 10** ^**4**^
	Rank	**1**	4	3	2	5

#### 4.7.2 Friedman test analysis

According to Table 12, the Friedman test results ($\chi^{2}(9)=53.5804$, *p* < 0.001) indicate that there is a significant difference in performance among the 10 algorithms tested on the 10 benchmark functions. Among them, ICOA ranks first with an average rank of 2.28, significantly better than other algorithms, and is the optimal algorithm in this experiment, with significant statistical advantages.

**Table 12:** Friedman test (CEC2020 D=30).

Function	ICOA	COA	SCA	WOA	HHO	WDO	PSO	APO	DBO	BKA
F1	**1.67**	2.00	8.00	3.93	9.00	6.00	4.97	7.00	2.43	10.00
F2	3.07	4.53	9.17	5.23	7.73	5.43	3.70	**2.87**	3.43	9.83
F3	**2.33**	5.80	6.40	7.30	8.80	2.93	5.23	3.63	2.57	10.00
F4	**3.40**	**3.40**	4.85	3.70	**3.40**	5.98	8.87	**3.40**	8.03	9.97
F5	1.80	4.67	7.57	7.10	9.60	4.20	3.57	**1.43**	5.80	9.27
F6	2.10	**2.07**	8.37	7.07	8.37	5.33	5.07	3.07	3.70	9.87
F7	2.13	4.70	7.53	6.93	9.47	4.30	3.73	**1.00**	5.80	9.40
F8	**1.63**	1.80	8.50	6.23	8.57	4.97	5.27	4.57	4.83	8.63
F9	**1.70**	1.90	6.10	6.50	9.20	4.93	7.97	3.37	3.80	9.53
F10	2.93	2.63	8.00	5.03	9.00	5.77	**1.63**	6.80	3.20	10.00
AVG	**2.28**	3.35	7.45	5.90	8.31	4.98	5.00	3.71	4.36	9.65
Rank	**1**	2	8	7	9	5	6	3	4	10

## 5 Results and analysis of engineering optimization experiments

To verify the optimization performance of the improved algorithm, a variety of typical engineering design problems were selected for solution and analysis. To more comprehensively demonstrate the feasibility and practicality of the ICOA algorithm in engineering optimization, the experiments employed the latest evaluation indicators and statistical analysis methods. First, the constraint-violation value *v* 25 was introduced in each engineering case to determine whether the obtained solution satisfies the imposed constraints; only after feasibility is ensured is the fitness value used to assess the quality of the solution. The experimental results were statistically analyzed and compared across different algorithms.

$$v=\frac{\sum_{i=1}^{p}\max(g_{i}(\bar{x}),0)+\sum_{j=p+1}^{m}\max(|h_{j}(\bar{x})|-\varepsilon ,0)}{m}$$ (25)

Specifically, each algorithm was independently executed 30 times on every engineering problem, and the following performance indicators were recorded: the best fitness value (Best), the mean fitness value (Mean), the standard deviation of fitness values (Std), the mean constraint violation (MV), and the feasibility rate (FR). Among them, MV represents the average constraint-violation value over the 30 independent runs, while FR denotes the proportion of runs in which the algorithm successfully produced feasible solutions.

The performance of algorithms on engineering constrained optimization problems was evaluated mainly using two indicators: the feasibility rate (FR) and the mean constraint violation (MV). FR measures how often an algorithm produces feasible solutions over multiple independent runs, with higher values indicating more consistent satisfaction of constraints. MV represents the average degree to which the solutions violate constraints, with lower values indicating better handling of constraints and fewer infeasible solutions. When algorithms have the same FR and MV, their performance is further compared based on the mean fitness value (Mean), where smaller values indicate better solutions. Overall, algorithms with higher FR, lower MV, and smaller Mean are considered more effective for solving engineering constrained optimization problems.

### 5.1 Tension/compression spring design problem

**Fig 12:**
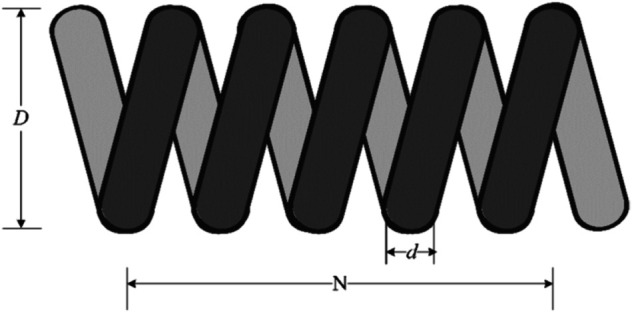
Schematic design of the tension/compression spring problem.

Designing extension/compression springs involves engineering optimization focused on reducing the weight of the spring. There are four design variables to optimize and four constraints to take into account, as shown in Fig 12. This optimization aims to minimize the weight of the spring by adjusting the values of D (mean coil diameter), d (wire diameter), and N (effective coil count). Using ICOA, the compression spring reaches an optimal weight of 0.0099657. Table 13 shows that ICOA achieves optimal results, demonstrating the superior effectiveness of ICOA.

**Table 13:** Experimental results for the tension/compression spring design problem.

Algorithm	Best	Mean	Std	Rank	d	D	N	FR	MV
ICOA	0.009975189	0.011157847	7.743732E-04	1	0.0500	0.3728	8.7022	1	0
COA	0.010265910	0.012569910	2.633947E-03	8	0.0513	0.4007	7.7482	1	0
SCA	0.010030252	0.011427895	7.363335E-04	4	0.0500	0.3734	8.7457	1	0
WOA	0.009924829	0.011190845	1.204329E-03	2	0.0503	0.3818	8.2654	1	0
HHO	0.010083397	0.011827684	1.415675E-03	5	0.0500	0.3690	8.9311	1	0
WDO	0.009901339	0.011895073	2.598764E-03	6	0.0500	0.3737	8.5978	1	0
PSO	0.009959001	0.012477084	6.017450E-03	7	0.0500	0.3722	8.7030	1	0
APO	0.010136243	0.025432322	4.357715E-02	9	0.0505	0.3835	8.4215	0.97	0.0083
DBO	0.009877197	0.011312251	1.990159E-03	3	0.0500	0.3743	8.5549	1	0
BKA	0.010422967	0.029897495	1.544123E-02	10	1.8861	0.5786	14.0113	1	0

Consider:

$$\vec{k}=\left[k_{1}\;\;k_{2}\;\;k_{3}\right]=\left[d\;\;D\;\;N\right]$$ (26)

Minimize:

$$f\left(\vec{k}\right)=\left(k_{3}+2\right)\times k_{2}\times k_{1}^{2}$$ (27)

Variable range:

$$\left\{ \begin{array}{c} 0.05⩽k_{1}⩽2.0\\ 0.25⩽k_{2}⩽1.3\\ 2.0⩽k_{3}⩽15.0 \end{array}\right.$$ (28)

Subject to:


$$g_{1}\left(\vec{k}\right)=1-\frac{k_{3}\times k_{2}^{3}}{71785\times k_{1}^{4}}⩽0\;g_{2}\left(\vec{k}\right)=\frac{4\times k_{2}^{2}-k_{1}\times k_{2}}{12566\times k_{1}^{4}}+\frac{1}{5108\times k_{1}^{2}}-1⩽0\;g_{3}\left(\vec{k}\right)=1-\frac{140.45\times k_{1}}{k_{2}^{2}\times k_{3}}⩽0\;g_{4}\left(\vec{k}\right)=\frac{k_{1}+k_{2}}{1.5}-1⩽0\;$$

As shown in Table 13, it can be inferred that ICOA exhibits d=0.05,D=0.3728 and *N* = 8.7022. The result shows a minimum weight of 0.0099657—0.28% better than that produced by COA. Compared with the other algorithms, ICOA achieves the best performance by producing the lowest spring weight, indicating its strong optimization capability.

### 5.2 Pressure vessel design problem

**Fig 13:**
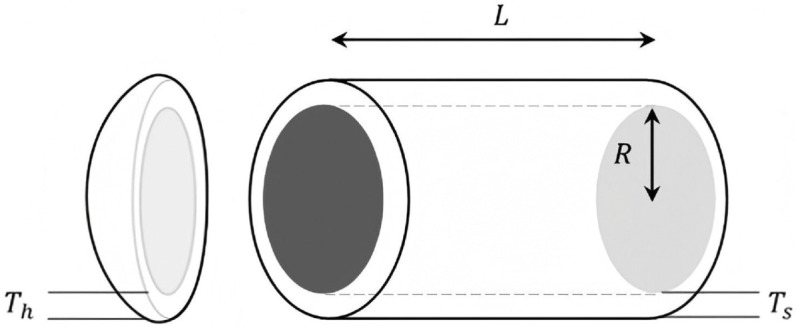
Schematic design of the pressure vessel problem.

This problem involves designing a cylindrical pressure vessel with hemispherical heads that can withstand a given internal pressure while minimizing the overall cost or weight. There are four key parameters to be optimized: shell thickness(*T*_*s*_), head thickness(*T*_*h*_), inner radius (R) and cylindrical length(L), as shown in Fig 13.

Consider:

$$\vec{k}=\left[k1,k2,k3,k4\right]=\left[T_{S}\;\;T_{H}\;\;R\;\;L\right]$$ (29)

Minimize:

$$f\left(\vec{k}\right)=0.6224k_{1}k_{3}k_{4}+1.7781k_{2}k_{3}^{2}+3.1661k_{1}^{2}k_{4}+19.84k_{1}^{2}k_{3}$$ (30)

Variable range:

$$\left\{ \begin{array}{c} 0⩽k_{1}⩽100\\ 0⩽k_{2}⩽100\\ 10⩽k_{3}⩽200\\ 10⩽k_{4}⩽200 \end{array}\right.$$ (31)

Subject to:


$$g_{1}\left(\vec{k}\right)=-k_{1}+0.0193k_{3}\leq 0\;g_{2}\left(\vec{k}\right)=-k_{2}+0.00954k_{3}\leq 0\;g_{3}\left(\vec{k}\right)=-\pi k_{3}^{2}k_{4}-\frac{4}{3}\pi k_{3}^{3}+1296000\leq 0\;g_{4}\left(\vec{k}\right)=k_{4}-240\leq 0\;$$

As shown in Table 14, it can be inferred that *T*_*s*_ = 0.8998, and *L* = 197.1797 for ICOA. The result shows a average weight of 7148.163262 —17.86% better than that produced by COA. Compared with other algorithms with average weight, ICOA is still the smallest. Consequently, it can be inferred that ICOA demonstrates an excellent optimization capability.

**Table 14:** Experimental results for the pressure vessel design problem.

Algorithm	Best	Mean	Std	Rank	$T_{S}$	$T_{H}$	R	L	FR	MV
ICOA	7256.1380	38677.8856	39614.4621	1	1.0464	0.5415	51.8170	95.2593	1	0
COA	6947.4733	67012.0239	42446.3325	7	0.8624	0.4550	41.5836	200.0000	1	0
SCA	7442.1755	46976.3730	44124.3308	3	0.9915	0.6878	49.9691	100.5631	1	0
WOA	6840.7469	69265.4841	39872.2057	8	1.1066	0.5681	57.3353	49.0453	1	0
HHO	6972.8170	73601.0920	39707.7954	9	1.1463	0.5904	58.6570	41.7205	1	0
WDO	8839.7513	61058.0515	45345.1529	6	1.3029	0.7669	66.3974	10.0000	1	0
PSO	7812.5778	48015.7391	37125.7735	4	1.0820	0.5529	52.4652	99.5944	1	0
APO	9303.4509	43562.4840	28631.1297	2	89.4809	55.6678	132.1030	157.5558	1	0
DBO	6052.1937	55188.7614	46462.4355	5	0.7913	0.3888	40.6749	200.0000	1	0
BKA	20768.3786	80791.9855	27026.0157	10	15.3002	4.0067	139.9687	50.9851	1	0

### 5.3 Three-bar truss design problem

**Fig 14:**
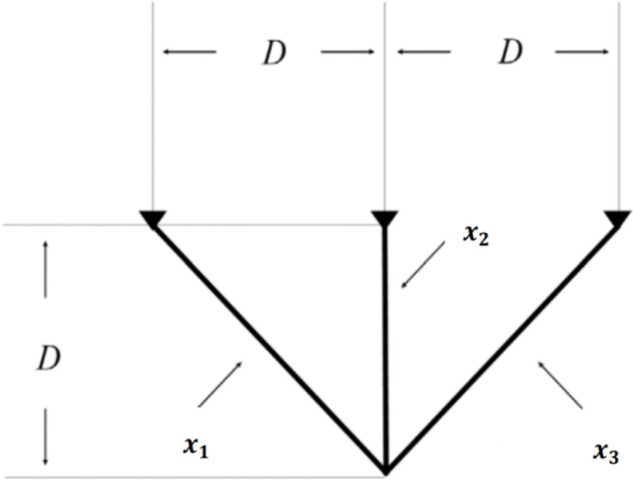
Schematic design for the three-bar truss problem.

This design is a fundamental structural configuration composed of three members arranged in a triangular form, which is widely used in bridges, roofs, and mechanical structures. As shown in Fig 14, the parameters include the dimensions, geometry and connection techniques of the rods to ensure that the structure meets the performance and cost goals within the given constraints.

Consider:

$$\vec{k}=\left[k_{1}k_{2}\right]$$ (32)

Minimize:

$$f\left(\vec{k}\right)=\left(2\sqrt{2}k_{1}+k_{2}\right)\times D$$ (33)

Variable range:


$$g_{1}\left(\vec{k}\right)=\frac{\sqrt{2}k_{1}+k_{2}}{\left(\sqrt{2}k_{1}^{2}+2k_{1}k_{2}\right)}P-\sigma \leq 0\;g_{2}\left(\vec{k}\right)=\frac{k_{2}}{\sqrt{2}k_{1}^{2}+2k_{1}k_{2}}P-\sigma \leq 0\;g_{3}\left(\vec{k}\right)=\frac{1}{\sqrt{2}k_{2}+k_{1}}P-\sigma \leq 0\;$$

Parameter:


$$D=100cm\;P=2kN/(cm^{2})\;\sigma =2kN/\left(cm^{2}\right)\;$$

As shown in Table 15, it can be inferred that *k*_1_ = 0.7880 and *k*_2_ = 0.4101 of ICOA. The result shows a minimum weight of 263.8971695. Compared to other algorithms, ICOA achieves the lowest minimum weight, showing its strong optimization ability.

**Table 15:** Experimental results for the triple bar truss design problem.

Algorithm	Best	Mean	Std	Rank	$k_{1}$	$k_{2}$	FR	MV
ICOA	263.8971695	263.9369567	0.030001957	1	0.7880	0.4101	1	0
COA	263.9312582	268.2321388	2.938286940	10	0.7890	0.4077	1	0
SCA	263.9829708	264.5931769	0.384943009	6	0.7944	0.3931	1	0
WOA	263.8968252	264.8007327	1.006862379	8	0.7876	0.4113	1	0
HHO	263.8958648	264.5718765	0.518070364	5	0.7888	0.4080	1	0
WDO	263.9121064	264.1155733	0.137020276	3	0.7925	0.3977	1	0
PSO	263.9807924	264.6539999	0.410625325	7	0.7971	0.3852	0.93	0.03
APO	263.9540301	264.3959055	0.381385810	4	0.1097	0.9759	1	0
DBO	263.8962011	263.9539950	0.054389925	2	0.7884	0.4090	1	0
BKA	264.1129752	265.0631604	0.713445034	9	0.6708	0.3032	1	0

### 5.4 Gear train design problem

**Fig 15:**
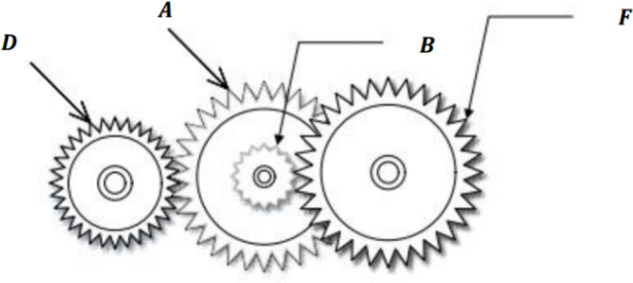
Schematic design of the gear train problem.

This problem involves selecting and arranging gears to achieve a desired speed ratio or torque transmission in mechanical systems. Fig 15 shows a gearing schematic with four gears: A, B, D, and F. The goal is to design a gear train that achieves a specified gear ratio while optimizing performance criteria such as size, weight, cost, and durability. The goal is smaller or lighter gear trains(*T*_*a*_,*T*_*h*_,*T*_*d*_,and*T*_*f*_) to reduce material costs and improve the efficiency of the system.

Consider:

$$\vec{k}=\left[k_{1}\;\;k_{2}\;\;k_{3}\;\;k_{4}\right]=\left[T_{a}\;\;T\;\;T_{d}\;\;T_{f}\right]$$ (34)

Minimize:

$$f\left(\overset{―}{k}\right)={\left(\frac{1}{6.931}-\frac{T_{b}T_{d}}{T_{a}T_{f}}\right)}^{2}$$ (35)

Variable range:

$$k_{i}=\{12,13,14,...,60\}\;i=1,2,3,4\;$$ (36)

As shown in Table 16, it can be inferred that *T*_*a*_ = 49,*T*_*b*_ = 13,*T*_*d*_ = 31. and *T*_*f*_ = 57 of ICOA. The minimum weight value is 9.93988E-11, which is 88.8% greater than the COA. Compared to the minimum weight values obtained by other algorithms, ICOA yields the smallest value. This shows that ICOA exhibits an effective optimization performance.

**Table 16:** Experimental results for the gear train design problem.

Algorithm	Best	Mean	Std	Rank	$T_{a}$	$T_{b}$	$T_{d}$	$T_{f}$	FR	MV
ICOA	9.93988E-11	2.61647E-09	3.27006E-09	1	49	13	31	57	1	0
COA	8.88761E-10	8.71899E-09	9.03519E-09	6	57	37	12	54	1	0
SCA	9.92158E-10	1.77105E-08	1.24244E-08	8	47	13	12	23	1	0
WOA	2.70086E-12	7.71679E-09	9.58533E-09	5	43	16	19	49	1	0
HHO	2.30782E-11	4.45970E-09	5.56535E-09	3	45	22	13	46	1	0
WDO	9.92158E-10	9.69937E-09	7.83049E-09	7	46	12	26	47	1	0
PSO	2.30782E-11	3.65914E-09	3.84626E-09	2	51	26	15	53	1	0
APO	2.70086E-12	6.43689E-08	8.61175E-08	9	53	41	59	40	1	0
DBO	9.93988E-11	5.64154E-09	7.88993E-09	4	57	13	31	49	1	0
BKA	2.35764E-09	2.04612E-06	1.99074E-06	10	19	36	12	43	1	0

### 5.5 Tubular column design

**Fig 16:**
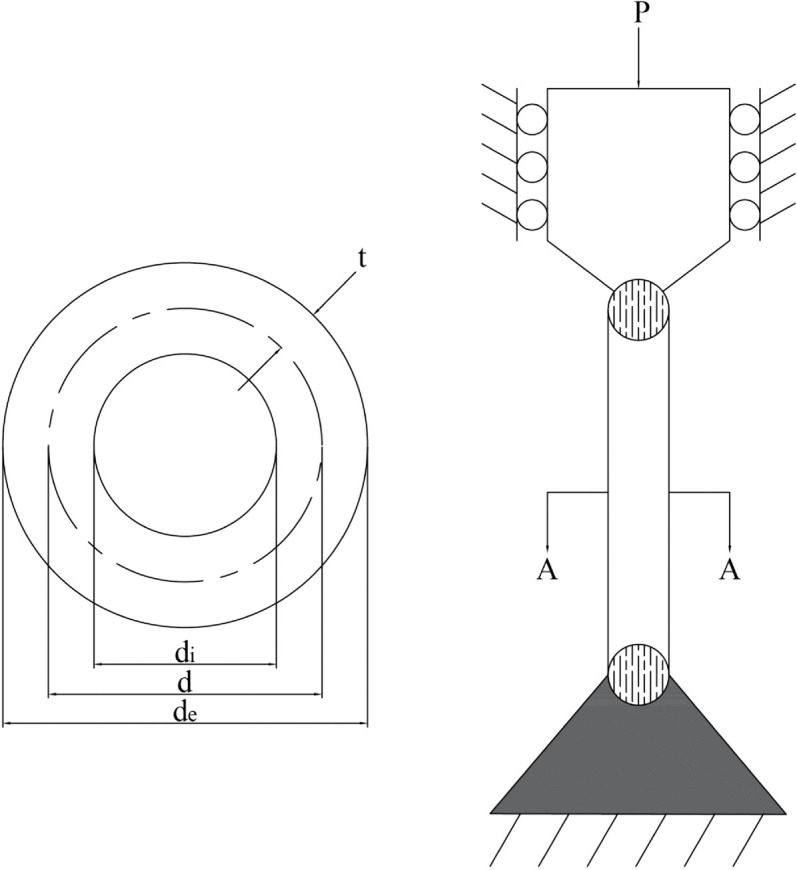
Schematic design of the tubular column problem.

The design problem involves a uniform tubular column subjected to compressive loads, with the aim of reducing the cost. As shown in Fig 16, it involves two variables: the column’s average thickness and the tube’s wall thickness. The structural column consists of a material with known yield strength and elasticity parameters.

Consider:

$$\vec{k}=\left[k_{1}\;\;k_{2}\right]=\left[d\;\;t\right]$$ (37)

Minimize:

$$f\left(\vec{k}\right)=9.8k_{1}k_{2}+2k_{2}$$ (38)

Variable range:

$$\{2\leq k_{1}\leq 14\;0.2\leq k_{2}\leq 0.8\;$$ (39)

Subject to:


$$g_{1}\left(\vec{k}\right)=1.59-k_{1}k_{2}\leq 0\;g_{2}\left(\vec{k}\right)=47.4-k_{1}k_{2}\left(k_{1}^{2}+k_{2}^{2}\right)\leq 0\;g_{3}\left(\vec{k}\right)=\frac{2.0}{k_{1}}-1\leq 0\;g_{4}\left(\vec{k}\right)=\frac{k_{1}}{14}-1\leq 0\;g_{5}\left(\vec{k}\right)=\frac{0.2}{k_{2}}-1\leq 0\;g_{6}\left(\vec{k}\right)=\frac{k_{2}}{8}-1\leq 0\;$$

As shown in Table 17, from which it can be concluded that *d* = 5.4519, *t* = 0.2922 of ICOA on the tubular column design problem. The result shows a minimum weight of 26.51741673—0.1% better than that produced by COA. Compared to the minimum weight values obtained by other algorithms, although the ICOA does not surpass DBO and WDO in overall performance, it still demonstrates a notable advantage in local search and specific subproblems. This indicates that ICOA demonstrates an effective optimization performance.

**Table 17:** Experimental results for the tubular column design problem.

Algorithm	Best	Mean	Std	Rank	*d*	*t*	FR	MV
ICOA	26.51741673	26.70621873	0.171360085	3	5.4519	0.2922	1	0
COA	26.54431225	26.85692397	0.207963746	6	5.4493	0.2930	1	0
SCA	26.72497195	27.00185603	0.163562116	7	5.4700	0.2945	1	0
WOA	26.49874434	27.11021984	0.508389291	9	5.4582	0.2913	1	0
HHO	26.49419813	26.79267770	0.274001934	5	5.4502	0.2920	1	0
WDO	26.50925902	26.62897213	0.062780923	2	5.4496	0.2923	1	0
PSO	26.51899642	26.74411416	0.123382181	4	5.4471	0.2927	1	0
APO	26.66265001	27.15271324	0.332646631	10	5.7041	0.5384	1	0
DBO	26.48638168	26.49158821	0.003692962	1	5.4520	0.2916	1	0
BKA	26.64014360	27.03797562	0.218192413	8	8.5826	0.4336	1	0

## 6 Discussion

The proposed Improved Crayfish Optimization Algorithm (ICOA) demonstrates significant advantages in solving engineering optimization problems. By incorporating a the initialization of the Sobol sequence, Lévy flight strategy, and a Euclidean distance–fitness balance competition mechanism, ICOA effectively addresses the imbalance between exploration and exploitation, thus enhancing global search capability and convergence efficiency. In the CEC2019 and CEC2020 benchmark function tests, ICOA consistently achieved faster convergence and higher solution accuracy across different dimensions, significantly outperforming COA and several other metaheuristic algorithms. Moreover, in multiple classical engineering optimization problems, ICOA also exhibited superior performance. For example, in the tension/compression spring design problem, pressure vessel design problem, three-bar truss design problem, gear train design problem, and tubular column design problems, ICOA outperformed COA with improvements up to 96.95, confirming its effectiveness and reliability in practical engineering applications.

Despite these promising results, the iterative update mechanism of ICOA still has certain limitations. First, while the combination of the Sobol sequence and Lévy flight enhances population diversity and the ability to escape local optima, it may also cause individuals to become overly dispersed in the search space, leading to instability in early-stage convergence. Second, although the Euclidean distance–fitness balance competition mechanism promotes the coordination between global and local search, its equilibrium is difficult to maintain in high-dimensional complex problems, which can result in either excessive exploration or insufficient exploitation. In addition, ICOA relies on interactive updates throughout the population, which substantially increases computational cost in large-scale problems and may slow down convergence in later iterations.

In future studies, efforts will focus on refining the ICOA update mechanism to improve its scalability and stability in large-scale problems, while also introducing adaptive parameter control to minimize dependence on manual tuning. Additionally, extending the algorithm to address multi-objective, dynamic, and uncertain optimization scenarios will further expand its applicability in complex engineering domains. Moreover, integration with deep learning and large models represents a promising direction, where data-driven learning of update rules and predictive guidance of search processes could significantly enhance the intelligence and cross-domain adaptability of ICOA.

## 7 Conclusion

To address the drawbacks of existing crayfish optimization algorithms, including slow convergence, susceptibility to local optima, and limited accuracy, an improved Crayfish Optimization Algorithm (ICOA) is proposed. Within ICOA lies a refined version of a crayfish-based optimization algorithm that leverages enhanced strategies for improvement. In the early stage, Sobol sequences are used to promote a more diverse population. In the foraging phase, the algorithm uses the Lévy flight strategy to avoid local optima and encourage a broader exploration of the solution space. In the competition phase, the foraging behavior and updating methods of crayfish individuals are sought by using the Euclidean distance-fitness balanced competition strategy to increase the information on location and fitness differences between individuals. Experimental results on CEC2019 and CEC2020 confirm the improved efficiency of the algorithm, demonstrating its performance across various test functions. Through rank test and statistical analyses, we demonstrate the substantial superiority of ICOA compared to its competitors in both CEC2019 and CEC2020 (with dimensions of 20, 30, and 50). Additionally, the practical significance of ICOA in engineering applications is further highlighted through the analysis of five engineering case studies. The results clearly confirm ICOA’s efficiency and strong performance on five optimization problems.

The proposed algorithm still has certain limitations. Although the Sobol sequence is adopted to replace random initialization for improving population uniformity and diversity, its advantage may be less significant in some discontinuous or highly multimodal optimization problems. Future research could consider combining the Sobol sequence with random perturbations or chaotic mappings to further enhance population diversity. In the Lévy flight strategy, parameter settings have a considerable impact on algorithm performance. Therefore, introducing an adaptive Lévy step-size mechanism that dynamically adjusts the step length according to fitness variation or iteration progress could improve stability and convergence efficiency. In addition, while the Euclidean Distance–Fitness Balanced Competition Strategy contributes to maintaining population diversity, it also increases computational complexity, since the distance matrix among all individuals must be computed in each iteration. Future work may explore cluster-based or density estimation-based competition mechanisms to reduce computational cost and improve overall algorithmic stability.

Future research will aim to extend the applications of the Enhanced Crayfish Optimization Algorithm (ECOA) to various fields, including function optimization, image processing, vehicle scheduling, and creating a multi-objective variant of ICOA, demonstrating its effectiveness in solving multi-objective tasks and providing diverse optimization results.

## Supporting information

S1 Dataset Minimum runnable. This folder contains the minimal runnable dataset required to reproduce the core analyses and results presented in this study. The data and scripts are designed to be executed in MATLAB.

S2 Dataset Raw data. This folder contains the original, unprocessed data collected for the experiments. These raw files serve as the basis for all data preprocessing and analysis steps described in the manuscript.
